# Molecular Engineering of Rigid Hydrogels Co-assembled from Collagenous Helical Peptides Based on a Single Triplet Motif

**DOI:** 10.1021/acsami.2c09982

**Published:** 2022-10-07

**Authors:** Santu Bera, Pierre-Andre Cazade, Shayon Bhattacharya, Sarah Guerin, Moumita Ghosh, Francesca Netti, Damien Thompson, Lihi Adler-Abramovich

**Affiliations:** † Department of Oral Biology, The Goldschleger School of Dental Medicine, Sackler Faculty of Medicine, The Center for Nanoscience and Nanotechnology, and The Center for the Physics and Chemistry of Living Systems, Tel Aviv University, Tel Aviv 6997801, Israel; ‡ Department of Physics, Bernal Institute, University of Limerick, Limerick V94T9PX, Ireland

**Keywords:** minimalist peptide design, collagen-mimetic, co-assembly, hybrid hydrogel, supramolecular packing

## Abstract

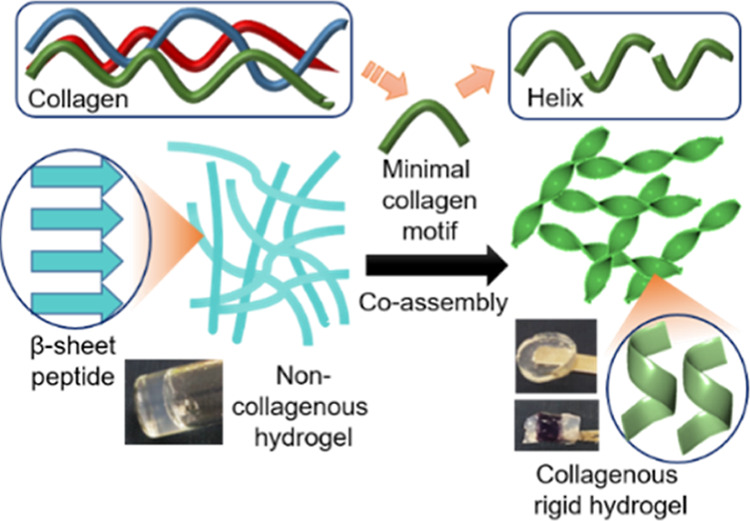

The potential of ultra-short peptides to self-assemble into well-ordered functional nanostructures makes them promising minimal components for mimicking the basic ingredient of nature and diverse biomaterials. However, selection and modular design of perfect *de novo* sequences are extremely tricky due to their vast possible combinatorial space. Moreover, a single amino acid substitution can drastically alter the supramolecular packing structure of short peptide assemblies. Here, we report the design of rigid hybrid hydrogels produced by sequence engineering of a new series of ultra-short collagen-mimicking tripeptides. Connecting glycine with different combinations of proline and its post-translational product 4-hydroxyproline, the single triplet motif, displays the natural collagen-helix-like structure. Improved mechanical rigidity is obtained *via* co-assembly with the non-collagenous hydrogelator, fluorenylmethoxycarbonyl (Fmoc) diphenylalanine. Characterizations of the supramolecular interactions that promote the self-supporting and self-healing properties of the co-assemblies are performed by physicochemical experiments and atomistic models. Our results clearly demonstrate the significance of sequence engineering to design functional peptide motifs with desired physicochemical and electromechanical properties and reveal co-assembly as a promising strategy for the utilization of small, readily accessible biomimetic building blocks to generate hybrid biomolecular assemblies with structural heterogeneity and functionality of natural materials.

## Introduction

Molecular self-assembly of evolved sequence-specific biopolymers plays a crucial role in the fabrication and functionalization of nanoscale structures in nature.^1−3^ Such dynamic materials can also be used to generate artificial nanostructures with tunable properties for a range of potential applications in regenerative medicine and nanotechnology.^4−6^ Peptides and proteins attract widespread attention due to their chemical versatility, biodegradability, and biocompatibility.^7−10^ It is well-established that the structure encodes the function in self-assembled nanostructures, yet an in-depth understanding of the relationships between molecular structures and macroscale properties remains elusive.^11,12^ Remarkably, by applying a reductionist approach, it has been revealed that ultra-short peptides, even those consisting of only two or three amino acid residues, can act as powerful self-assembly motifs.^13,14^ Deciphering the mechanisms underlying peptide self-assembly in minimal systems could uncover the assembly principles of constituent complex proteins and facilitate the modular design of easily tailorable, inexpensive, and eco-friendly materials with desired functions.

While natural peptides and proteins can adopt two distinct atomic-level structural arrangements, namely, β-sheet and α-helix, the self-assembled hierarchical architectures created by ultra-short peptides are predominantly β-sheet.^15,16^ On the other hand, collagen, the most abundant protein in mammals and the main source of structural strength of connective tissues, is helical.^17−19^ The three peptide strands comprising collagen adopt a polyproline type II helical conformation and intertwine around a common axis into a left-handed helical symmetry to form the characteristic collagen triple helix. This is achieved by two important features: the presence of a glycine (Gly) residue at every third position in the sequence and the presence of a high concentration of imino acids that contain both imine (>C=NH) and carboxyl [−C(=O)–OH] functional groups.^19^ The triplet Gly–Xaa–Yaa is frequently found as a repeating sequence in collagen, where Xaa and Yaa are commonly proline (Pro) and its post-translationally modified product, 4-hydroxyproline (Hyp), respectively.^19^ Thus, (Gly–Pro–Hyp)_*n*_ is represented as the signature motif in the amino acid sequence of collagen.

Collagen has long been the target of biomimetic design due to the numerous challenges related to the extraction, characterization, and use of natural collagen.^20^ In 1994, the first crystal structure of the collagen model peptide (Pro–Hyp–Gly)_4_–(Pro–Hyp–Ala)–(Pro–Hyp–Gly)_5_ was reported to organize into polyproline II helices, with supercoiling and interchain hydrogen bonding, following the Rich and Crick model.^21^ Many collagen model peptides, including (Gly–Pro–Hyp)_9_ and (Pro–Hyp–Gly)_10_, show the triple-strand helical twist characteristic of collagen.^22,23^ The sequence–stability relation of collagen structure has been widely investigated. In particular, the post-translational hydroxylation converting Pro into Hyp is assumed to be essential for providing stability to the collagen triple helix structure through the hydrogen-bonded network formation with encapsulated water molecules.^24^ The thermal stability of triple-helical collagen is reported to be improved by the hydroxyl group on the pyrrolidine ring of the Hyp residues as demonstrated for (Pro–Pro–Gly)_10_ (25 °C) *versus* (Pro–Hyp–Gly)_10_ (58 °C).^24^ However, this hydration-based stabilization model has been challenged by other studies. For example, replacing the central Hyp of (Pro–Hyp–Gly)_10_ with (2*S*,4*R*)-4-fluoroproline (Flp) was found to greatly increase collagen stability due to favorable inductive effects.^25^ Similarly, a number of collagen-mimicking peptide sequences without Hyp, such as (Pro–Pro–Gly)_10_ and (Pro–Pro–Gly)_9_, have also been shown to adopt a helical conformation.^26−28^ These studies highlight the contradictions surrounding the role of Hyp in facilitating a helical organization.

To date, long (>9 residue) peptides have been required to attain the helical collagen-like conformation, and most *de novo*-designed collagen-mimetic sequences contain two or more triplet repeats. Some of these peptides exhibit higher-order self-assembly and thus have potential for use in biomedical applications.^29−31^ However, the discrete characteristic levels of collagen hierarchical self-assembly (helix, nanofiber, and hydrogel) are very rarely reported within the same system.^20^ Therefore, there is an unmet need for minimal motifs capable of achieving the desired structure and function of collagen. A novel strategy involving multicomponent supramolecular co-assembly of simple building blocks has recently emerged as a promising approach to fabricate programmable multiscale hierarchical hybrid biomaterials with desired functionalities.^32−36^ This approach can produce dynamically assembled hybrid materials that possess complexity and functionality, which would not be achievable by single components. Most importantly, the co-assembly of selected biomolecules with different chemical structures and physical properties can produce hybrid biomaterials with precisely tailored functions derived from a synergistic combination of properties of individual building units.

Here, we performed sequence engineering of Fmoc–Gly–Pro–Hyp, a single collagen tripeptide repeating unit with N-terminal modification,^37^ which adopts a left-handed polyproline II conformation. Our systematic design strategy allowed us to investigate the significance of each amino acid residue and examine the importance of their relative position in the sequence, across a series of ultra-short collagen-mimicking tripeptides, Fmoc–Hyp–Pro–Gly, Fmoc–Gly–Pro–Pro, and Fmoc–Pro–Pro–Gly. We demonstrate that all three modified tripeptides adopt a polyproline II helical strand structure similar to that of the parent tripeptide Fmoc–Gly–Pro–Hyp,^37^ providing evidence for the role of the hydroxyl group of Hyp in promoting triple helix stabilization rather than single helix formation. Supramolecular co-assembly of these collagen-mimicking peptides with a β-sheet-forming dipeptide, Fmoc–diphenylalanine (Fmoc–Phe–Phe), produces hybrid hydrogels with distinct properties, completely different from those of the Fmoc–Phe–Phe self-assembled hydrogel. The hybrid hydrogels adopt polyproline II helicity and produce twisted fibrils with significantly improved mechanical rigidity compared to that of the dipeptide co-former, due to favorable supramolecular interactions. Using atomistic molecular dynamics (MD) simulations, we show that these improvements are a result of the co-assembly between Fmoc–Phe–Phe and the two tripeptides containing adjacent proline moieties (Fmoc–Gly–Pro–Pro and Fmoc–Pro–Pro–Gly), which produces a stronger and more tightly packed π–π stacking network than Fmoc–Phe–Phe with Fmoc–Hyp–Pro–Gly. Our molecular engineering approach produces robust hybrid hydrogels with collagenous polyproline II features through simple supramolecular co-assembly of non-collagenous hydrogel building blocks. The current findings help pave the way for the modular design of a diverse class of complex biological and bio-inspired materials for sustainable technologies.^38,39^

## Materials and Methods

### Materials

All the peptides reported here were synthesized by DG Peptides Co. Ltd. (Hangzhou city, Zhejiang province, China). After purification (>95%, confirmed by HPLC), mass spectrometry was employed to confirm the identity of peptides. Peptides were kept at low temperature (−20 °C).

### Preparation of Peptide Assemblies

For assembly, first, dimethyl sulfoxide (DMSO) was used to prepare a highly concentrated (100 mg/mL) solution of peptides by vigorous vortexing. Then, this solution was used as stock to prepare a dilute solution of desired concentration by using double distilled water. For most of the studies, a final peptide concentration of 5 mg/mL was used. Aging was performed through continuous shaking of all final solutions for 48 h. Peptide solutions were then allowed to completely self-assemble by storing at 18 °C for 7 days.

### Hydrogel Preparation

Hydrogels were prepared according to the procedure described in our earlier study.^37^ A 5 mg/mL final concentration of peptide was used for the gelation. Briefly, 100 mg/mL stock solution of Fmoc–Phe–Phe and all the collagen-mimetic tripeptides were prepared in DMSO through vigorous vortexing. For single-component hydrogel, 50 μL of stock solution was mixed with 950 μL of double distilled water (DDW) through continuous vortexing. For co-assembly systems, the two peptide stock solutions were mixed at a volume ratio of 2:1 (33.4 and 16.6 μL) for Fmoc–Phe–Phe and tripeptide, respectively. 950 μL of DDW was then added to dilute the solutions to final concentrations of 5 mg/mL.

### Fourier Transform Infrared Spectroscopy

KBr infrared cards purchased from Sigma-Aldrich, Rehovot, Israel, were used to measure the Fourier transform infrared (FTIR) spectra of samples. After complete self-assembly of peptide solution/hydrogel, these were drop casted (30 μL) on KBr cards and fully dried using vacuum. To avoid signal from water, saturation with D_2_O was performed two times and the samples were dried completely under vacuum. FTIR spectra were collected using a nitrogen-purged Nicolet Nexus 470 FTIR spectrometer (Nicolet, Offenbach, Germany) equipped with a deuterated triglycine sulfate detector by following the method described in our previous study.^14^

### CD Spectroscopy

5 mg/mL final peptide solutions were prepared in DMSO/DDW as described earlier and used for circular dichroism (CD) measurement after the final self-assembly process. The hydrogels were prepared according to the procedures described above. All the hybrid hydrogels and bovine collagen solution type I from Sigma were also analyzed by CD. CD spectra were recorded using a Chirascan spectrometer (Applied Photophysics, Leatherhead, UK) fitted with a Peltier temperature controller set to the desired temperature, using methods described in details in our previous report.^14^ For temperature-dependent CD analysis, hybrid hydrogels of 5 mg/mL concentration were spread between demountable quartz cuvettes with an optical path length of 0.1 mm. The range of temperature was 5–80 °C. For a particular temperature, data were acquired after 10 min of reaching that temperature. The change in the 227 nm peak position was plotted against the temperature. Each experiment was performed in triplicate, and the average value was plotted.

### Transmission Electron Microscopy

Samples were prepared by drop-casting 5 μL of peptide solutions on 400 mesh copper grids and allowed to absorb for 1 min. After that, a bloating paper was utilized to remove the excess liquids from the grid. 2% uranyl acetate was used for negative staining. Samples were examined in a JEOL 1200EX electron microscope operating at 80 kV.

### Rheological Analysis

Rheological analysis of approximately 200 μL of hydrogels was performed using an AR-G2 rheometer (TA Instruments, USA) according to the procedure described in detail in our previous study.^37^

## Results and Discussion

### Rationale of the Peptide Design

**Figure 1:**
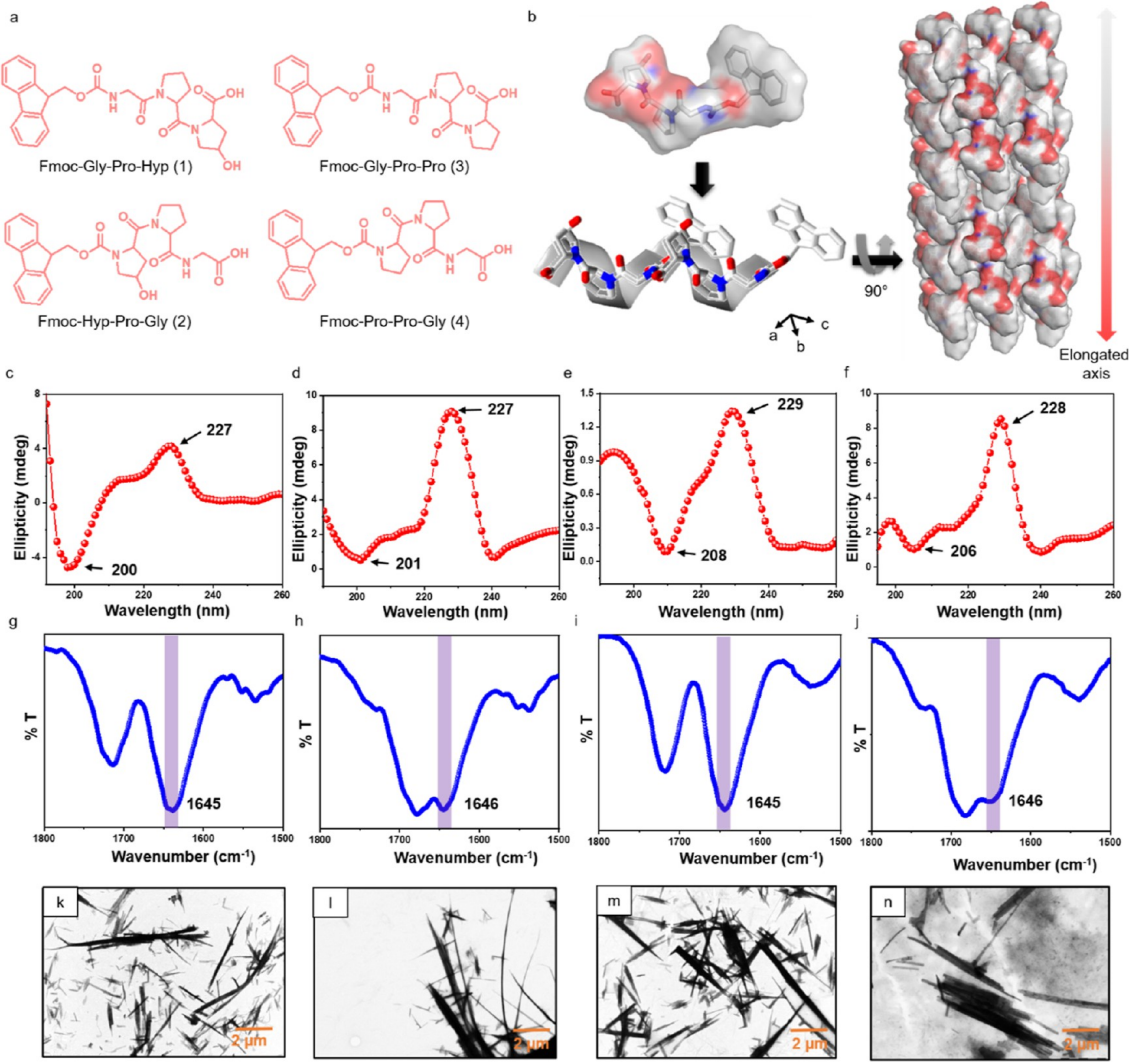
Molecular engineering of collagen-mimicking helical peptide sequences. (a) Chemical structures of the tripeptides: (1) Fmoc–Gly–Pro–Hyp, (2) Fmoc–Hyp–Pro–Gly, (3) Fmoc–Gly–Pro–Pro, and (4) Fmoc–Pro–Pro–Gly. (b) Single-crystal structure of Fmoc–Gly–Pro–Hyp showing formation of collagen-like helix structures, which stack through intermolecular hydrogen bonding and directional aromatic zipper-like packing. Cambridge Crystallographic Data Centre (CCDC) ref. no. 1962894.^37^ (c–f) CD spectra of (c) Fmoc–Gly–Pro–Hyp, (d) Fmoc–Hyp–Pro–Gly, (e) Fmoc–Gly–Pro–Pro, and (f) Fmoc–Pro–Pro–Gly. (g–j) FTIR spectra of (g) Fmoc–Gly–Pro–Hyp, (h) Fmoc–Hyp–Pro–Gly, (i) Fmoc–Gly–Pro–Pro, and (j) Fmoc–Pro–Pro–Gly. (k–n) HRTEM images of (k) Fmoc–Gly–Pro–Hyp, (l) Fmoc–Hyp–Pro–Gly, (m) Fmoc–Gly–Pro–Pro, and (n) Fmoc–Pro–Pro–Gly. (k–n) Scale bar = 2 μm.

The design of the parent tripeptide, Fmoc–Gly–Pro–Hyp (1, Figure 1a), was based on the abundance of the Gly–Pro–Hyp triplet in the sequence of native collagen.^37^ In order to investigate the role of the amino acid order in the peptide sequence in the structure formation and function of the minimal collagen model peptide, we first designed the reverse tripeptide, Fmoc–Hyp–Pro–Gly (2). Next, to resolve the debate concerning whether the Hyp group is indeed essential for the helical conformation of the backbone of a minimalistic model peptide, we excluded Hyp and designed the Fmoc–Gly–Pro–Pro peptide (3). Finally, again aiming to examine the importance of amino acid relative position, we reversed the order of peptide 3 and designed the Fmoc–Pro–Pro–Gly tripeptide (4).

### Molecular Conformation and Self-Assembled Structure of the Tripeptides

The single-crystal structure of Fmoc–Gly–Pro–Hyp revealed that the backbone adopted a left-handed polyproline II helical conformation similar to the conformation of a single strand of collagen (Figures 1b and S1).^37^ This conformation was completely different from the previously identified α-helical structure of hydrophobic tripeptides with an N-terminal Gly residue (Table S1).^40,41^ The polyproline II helical conformation in solution was confirmed by the characteristic peaks of CD spectra, with a negative peak at ∼200 nm and a positive broad maxima in the range of 220–230 nm (Figure 1c).^20^ The CD spectra of the three modified tripeptides, Fmoc–Hyp–Pro–Gly, Fmoc–Gly–Pro–Pro, and Fmoc–Pro–Pro–Gly, showed the maximum at 227, 229, and 228 nm, and the minimum around 201, 208 and 206 nm, respectively, emphasizing the presence of a polyproline type II helix (Figure 1d–f). Concentration-dependent CD analysis revealed an increase of the negative maxima as the concentration was elevated, indicative of self-assembly in solution (Figure S2). FTIR spectroscopy of the native peptide, Fmoc–Gly–Pro–Hyp, revealed an amide I band at ∼1646 cm^–1^ with a shoulder at around 1700 cm^–1^, indicating that the predominant secondary structure was a polyproline II helix (Figure 1g). Similar FTIR data of the three modified tripeptides (2–4) revealed distinct vibrational frequencies at 1646, 1645, and 1646 cm^–1^, respectively, in the amide I region, again indicating a predominantly polyproline II helical conformation in the dried samples, a topography precisely matching the structure in solution (Figure 1h–j). Therefore, all the tested tripeptides could adopt a polyproline II helical backbone conformation irrespective of the sequence order of the amino acid and the presence or absence of the Hyp motif. This clearly demonstrates the strong and broad preference of the Gly–Xaa–Yaa triplet to fold into a polyproline II helical conformation, which is not affected by rearranging the amino acid sequence and may therefore be generic in nature. In addition, the results indicate that the contribution of the −OH group in Hyp is not essential for backbone orientation into a polyproline II helical structure. A control peptide without an N-terminal Gly residue, Fmoc–Leu–Pro–Hyp, showed the presence of a disordered structure rather than polyproline conformation, clearly demonstrating the requirement of the Gly residue at the terminal position to acquire such a conformation by a single triplet motif (Figure S3).

The polyproline II helical secondary structure of all the designed tripeptides inspired us to study the macroscale self-assembly properties of the four peptides in solution. High-resolution transmission electron microscopy (HRTEM) imaging revealed the formation of similar elongated crystalline needle-type structures for all four tripeptides (Figure 1k–n).

### Fabrication of Multi-component Hybrid Hydrogels

**Figure 2:**
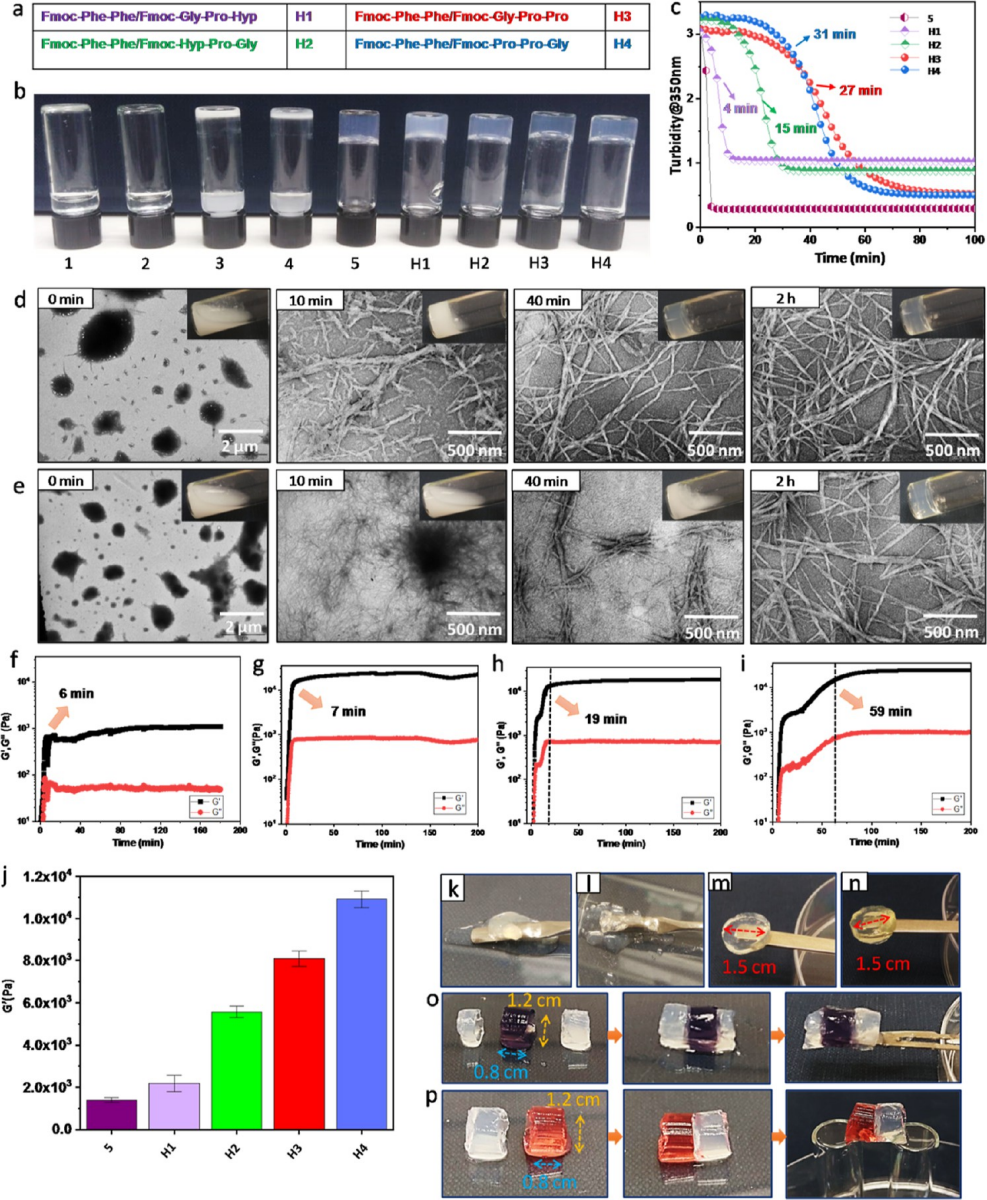
Co-assembled hybrid hydrogels of collagen-mimicking peptides and Fmoc–Phe–Phe. (a) The four hybrid systems: (H1) Fmoc–Phe–Phe/Fmoc–Gly–Pro–Hyp, (H2) Fmoc–Phe–Phe/Fmoc–Hyp–Pro–Gly, (H3) Fmoc–Phe–Phe/Fmoc–Gly–Pro–Pro, and (H4) Fmoc–Phe–Phe/Fmoc–Pro–Pro–Gly. (b) Gelation demonstrated by inverted vials of individual building blocks and hybrid hydrogels: (1) Fmoc–Gly–Pro–Hyp, (2) Fmoc–Hyp–Pro–Gly, (3) Fmoc–Gly–Pro–Pro, (4) Fmoc–Pro–Pro–Gly, (5) Fmoc–Phe–Phe, (H1) Fmoc–Phe–Phe/Fmoc–Gly–Pro–Hyp, (H2) Fmoc–Phe–Phe/Fmoc–Hyp–Pro–Gly, (H3) Fmoc–Phe–Phe/Fmoc–Gly–Pro–Pro, and (H4) Fmoc–Phe–Phe/Fmoc–Pro–Pro–Gly. (c) Turbidity measured at 350 nm over time. (d,e) Time-lapse optical images and corresponding TEM images of (d) H2 and (e) H4. (f–i) Time sweep oscillatory measurements of *in situ* hydrogel formation by (f) H1, (g) H2, (h) H3, and (i) H4. (j) Comparison of the mechanical rigidity of Fmoc–Phe–Phe and hybrid hydrogels at the frequency of 10 rad/s. The data represent the mean ± std error for *n* = 3 independent experiments. (k–n) Self-supporting properties of the hybrid hydrogels: (k) H1, (l) H2, (m) H3, and (n) H4. (o,p) Self-healing properties of (o) H3 and (p) H4.

Attempts to produce a hydrogel revealed that the designed collagen-mimetic short peptides formed organized nanostructures but remained in the solution state (Figures 2 and S4). Fmoc–Gly–Pro–Hyp and Fmoc–Hyp–Pro–Gly produced a clear solution without any detectable change even after several weeks. On the other hand, Fmoc–Gly–Pro–Pro and Fmoc–Pro–Pro–Gly showed the formation of a turbid solution immediately after the addition of water. Upon longer incubation, the peptide precipitated at the bottom of the vial leaving the solution clear. This inability to form a hydrogel and mimic the full higher-order assembly of natural collagen (helix to nanofibers to hydrogel) is characteristic of most of the previously designed biomimetic collagen-like short peptide helices.^20,42^ We, therefore, utilized the co-assembly approach, using the Fmoc–Phe–Phe (5) dipeptide as the co-assembly partner (Figure 2a). Fmoc–Phe–Phe can self-assemble into nanofibers and form macroscopic, self-supporting hydrogels under physiological conditions (Figure 2b).^43,44^ Based on its molecular self-assembly, Fmoc–Phe–Phe has been extensively utilized to incorporate bioactive ligands *via* supramolecular co-assembly into next-generation hybrid hydrogel scaffolds, combining the chemical and physical properties of the respective components.^45−47^ We, therefore, explored whether the co-assembly of each of our designed tripeptides with Fmoc–Phe–Phe could generate hybrid hydrogels that possess the advantageous properties of both components, in this case the collagen-mimetic structure of the tripeptides and the rigidity of the non-collagenous Fmoc–Phe–Phe hydrogelator.

Single or multicomponent gelation was carried out using the solvent-switch method in 5% DMSO solution in water. After screening several combinations, we selected a 2:1 molar ratio of Fmoc–Phe–Phe/Fmoc–tripeptide as optimal for the co-assembly of the hydrogel system as these conditions showed the best results with co-assembly of Fmoc–Phe–Phe with all four Fmoc–tripeptides (1–4) producing stable, transparent, and self-supporting hydrogels that resembled the hydrogel produced from the single-component Fmoc–Phe–Phe (Figure S5). To examine the kinetics of the co-assembly process, the absorbance spectra at 350 nm were measured with respect to time for different systems and compared the results with the spectrum of Fmoc–Phe–Phe (Figure 2c). While the absorbance of Fmoc–Phe–Phe solution rapidly decayed to an optical density (OD) of 0.298 within 5 min, the hybrid hydrogels exhibited much slower kinetic profiles. The OD of the Fmoc–Phe–Phe/Fmoc–Gly–Pro–Hyp (H1) and Fmoc–Phe–Phe/Fmoc–Hyp–Pro–Gly (H2) hybrid systems started to decrease at 4 and 15 min, respectively, and reached a plateau of ODs 1.052 and 0.880 after 11 and 29 min, respectively, consistent with the opaqueness of the final gels. The two other hybrid systems, namely, Fmoc–Phe–Phe/Fmoc–Gly–Pro–Pro (H3) and Fmoc–Phe–Phe/Fmoc–Pro–Pro–Gly (H4), required even longer times of 53 and 57 min to reach OD of 0.511 and 0.561, respectively, and form transparent hydrogels (Figure 2c).

The kinetics of the co-assembly was further investigated by recording optical images of the gels over time and monitoring the underlying nanostructures through time-lapse transmission electron microscopy (TEM, Figures 2 and S6). The starting turbid solution of pristine Fmoc–Phe–Phe gradually cleared over 10 min as the self-assembly process was completed, producing an optically transparent gel (Figure S6). In contrast, the times required for the turbid solutions of the four hybrid systems to become transparent gels were 25, 40, 105, and 120 min for H1–H4, respectively. The discrepancy between the time scales of the vial turbidity assay and the OD at 350 nm is probably because of the different volumes of solution used in the different methods. At time point zero, TEM imaging of single-component Fmoc–Phe–Phe as well as all four hybrid systems showed large spherulite structures capable of scattering light, which explains the high OD of the solution (Figures 2d and S6). These spherulite structures then served as nucleation seeds to produce an entangled fibrous network at different times depending on the system. The significant difference in the kinetics of gel formation seen between the single and hybrid systems indicates that the dynamic co-assembly process in the two-component systems is controlled by the different intermolecular interactions.

Rheological analysis was employed to characterize the gel formation and the mechanical properties of the hybrid hydrogels. Oscillatory time sweep measurements over 200 min, performed at a fixed strain of 0.1% and a frequency of 1 Hz, revealed that the storage modulus reached a plateau indicating completion of gel formation after 2 min for Fmoc–Phe–Phe and after 6, 7, 19, and 59 min for the four hybrid hydrogels (H1–H4), respectively (Figures 2f–i and S7). These gel formation times are in good agreement with the results of the other kinetic experiments. Dynamic frequency sweep experiments (Figure S7) demonstrated that the *G*′ and *G*″ values of all the assemblies were independent of the frequency, confirming their gel-like behavior. In addition, the elastic modulus *G*′ exceeded the viscous modulus *G*″ in all cases, indicating that the gels were primarily elastic rather than viscous in nature. The values of the storage modulus at a specific frequency of 10 rad/s were 1395, 2185, 5572, 8089, and 10 918 Pa for Fmoc–Phe–Phe and the four hybrid hydrogels, H1–H4, respectively (Figure 2j). These findings indicate a significant improvement in the mechanical rigidity of the hybrid hydrogels prepared through incorporating the three new designed tripeptides, with an increase as large as 1 order of magnitude obtained for H4. The results of dynamic strain sweep experiments (at 1 Hz frequency) over a range of 0.01–200% strain showed a wide linear viscoelastic region (LVR) for all the hybrid hydrogels, demonstrating the formation of a stable gel similar to the native Fmoc–Phe–Phe (Figure S8). All the hydrogels showed an LVR at up to 20% strain. Finally, we used step strain experiments to examine the recovery of the mechanical properties of the gels after shear deformation (Figure S9). In common with pristine Fmoc–Phe–Phe, all the hybrid gels exhibited sheer recovery properties over multiple cycles. Visual examination of the self-standing properties of the two-component gel samples revealed a clear correlation with their mechanical rigidity (Figure 2k–n). To visually demonstrate the self-healing capability of the hybrid hydrogels, discrete blocks of gel were held together in contact for 3–5 min, and the pieces were observed to merge into a stable self-supporting cylinder (Figure 2o,p). These fused cylinders could be suspended in air just by holding one side. This demonstrates that co-assembly confers a significant advantage with respect to the robustness of the gels and gelation kinetics, without impairing the sheer recovery ability of the parent Fmoc–Phe–Phe hydrogel.

### Inducing Collagenous Properties in Hybrid Hydrogels

**Figure 3:**
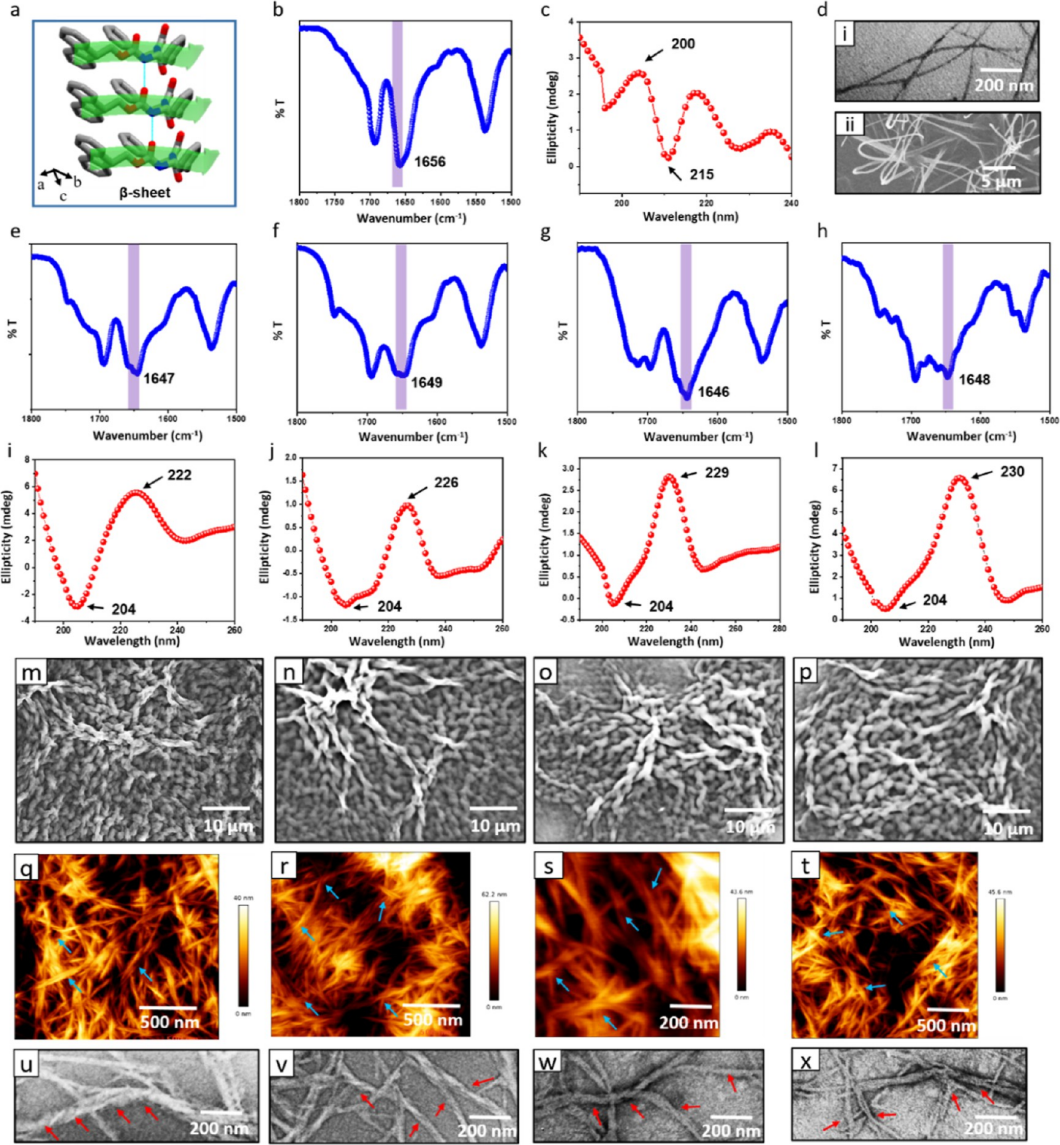
Embedding helicity into co-assembly of hybrid hydrogels. (a) β-sheet-like crystal packing of Fmoc–Phe–Phe. CCDC ref. no. 1027570.^48^ (b) FTIR and (c) CD spectra of Fmoc–Phe–Phe. (d) (i) TEM and (ii) SEM images of Fmoc–Phe–Phe. (e–h) FTIR spectra of the hybrid hydrogels H1–H4. (i–l) CD spectra of the hybrid hydrogels. (m–p) SEM images of the hybrid hydrogels. (q–t) AFM images of the hybrid hydrogels. (u–x) TEM images of the hybrid hydrogels. (e,i,m,q,u) H1. (f,j,n,r,v) H2. (g,k,o,s,w) H3. (h,l,p,t,x) H4. The thicker fibrils formed by co-assembly (30–40 nm) compared to just Fmoc–Phe–Phe alone [∼10 nm, panel d(i)] are marked by the blue and red arrows in panels (q–x).

The single-crystal structure of Fmoc–Phe–Phe exhibits a parallel β-sheet conformation composed of the molecular units (Figure 3a).^48^ FTIR and CD spectroscopic analyses were carried out to examine the backbone secondary structures of the molecular units within the Fmoc–Phe–Phe hydrogels. The FTIR spectra of the dried hydrogel contain two peaks at 1656 and 1692 cm^–1^, which arise from the imperfect stacking of the amide and carbamate groups, respectively, of the Fmoc moiety (Figure 3b).^49^ The CD spectra for the hybrid hydrogels showed positive and negative Cotton effects at 200 and 215 nm, respectively, confirming a β-sheet-rich structure (Figure 3c).^43^ TEM and scanning electron microscopy (SEM) images revealed that β-sheet stacking produced a dense network of thin, straight fibers with diameters on the order of tens of nanometers (Figure 3d). By contrast, the amide I region of the FTIR transmittance spectra of all four hybrid hydrogels exhibited similar sharp peaks at 1647, 1649, 1646, and 1648 cm^–1^ for H1–H4, respectively, with the additional distinct peak at ∼1690 cm^–1^, suggesting a transition from β-sheet to polyproline II conformation in the two-component hydrogels (Figure 3e–h) and confirming the structural modulation caused by the co-assembly of Fmoc–Phe–Phe with the collagen-mimicking peptides. CD spectra provided further support for the helical secondary structure of the co-assemblies (Figure 3i–l). The spectral patterns of the hybrid systems were very different from that of the native Fmoc–Phe–Phe hydrogel, showing spectral profile characteristics of a polyproline II helical conformation with well-defined negative peaks at ∼204 nm and positive peaks between 220 and 230 nm observed for all four hybrid hydrogels.

To further verify the presence of a polyproline II helical conformation, thermal unfolding experiments were carried out to observe the cooperative transition by monitoring the shift of the spectral maximum with increasing temperature (Figure S9). When melting experiments were performed in the range of 5–90 °C, all the hybrid hydrogels exhibited a cooperative transition of the peak at ∼225 nm in the melting profile, with a major transition between 40 and 42 °C. This finding of a similar melting temperature and thermal unfolding profile confirms the presence of a collagen-helix conformation in the hybrid systems. Therefore, we deduce that intermolecular hydrogen bonds (HBs) and π–π aromatic interactions between the two types of building units govern the final three-dimensional architecture of the hybrid systems.

CD and FTIR experiments were also performed for native collagen to confirm the similarities of the secondary structures of type I collagen and the hybrid hydrogels (Figure S10). FTIR analysis of native collagen showed the signature amide I band at 1650 cm^–1^ for polyproline II helical organization.^20^ The CD spectrum revealed the characteristic negative peak at ∼201 nm and a positive broad maxima in the range of 220–230 nm, signifying a polyproline II helical conformation.^20^ These results further confirmed the secondary structure obtained for designed hybrid hydrogels to be similar to native collagen. However, an attempt to investigate gelation by native collagen showed that it could not form a hydrogel under similar concentrations (Figure S10). The rheological time sweep experiment further confirmed the inability of gel formation by native collagen (Figure S10).

Having verified the polyproline II helical nature of the two-component hydrogel systems, the next step was to understand whether the nanoscale helicity encoded in the self-assembled structures was also manifested at the macroscale. SEM, atomic force microscopy (AFM), and TEM imaging revealed that the helicity of the co-assembly is preserved in the fibril networks formed at the scale of tens of microns, demonstrating the hierarchical nature of the packing conferred by co-assembly with the molecular structures of the peptides dictating the nanoscale supramolecular packing, which is in turn faithfully translated at the micron scale. SEM images showed that the thinner, straight fibers of the pristine Fmoc–Phe–Phe hydrogel were transformed after co-assembly into several-micrometer long, thicker, twisted fibrils (Figure 3m–p), with a significantly larger lateral thickness compared to Fmoc–Phe–Phe. Visualization of a larger area under lower magnification indicated that these self-assembled nanofibers permeated uniformly throughout the sample. AFM images provided additional support for the SEM observations. The assimilation of helical twist with increased fiber thickness of 30–40 nm was identified for all hybrid hydrogels, in contrast to the ∼10 nm thick straight fibers of pristine Fmoc–Phe–Phe (Figure 3q–t). Twisted fibers with a clearly distinguishable helical pitch were also observed in TEM images of the hybrid systems (Figure 3u–x). Therefore, the emergence of thickened, twisted architecture of the nanofibers suggests that strong non-covalent interactions between the building components produce robust co-assembled hybrid systems.

### Confirming the Two-Component Co-assembly by Time-of-Flight Secondary Ion Mass Spectrometry Analysis

**Figure 4:**
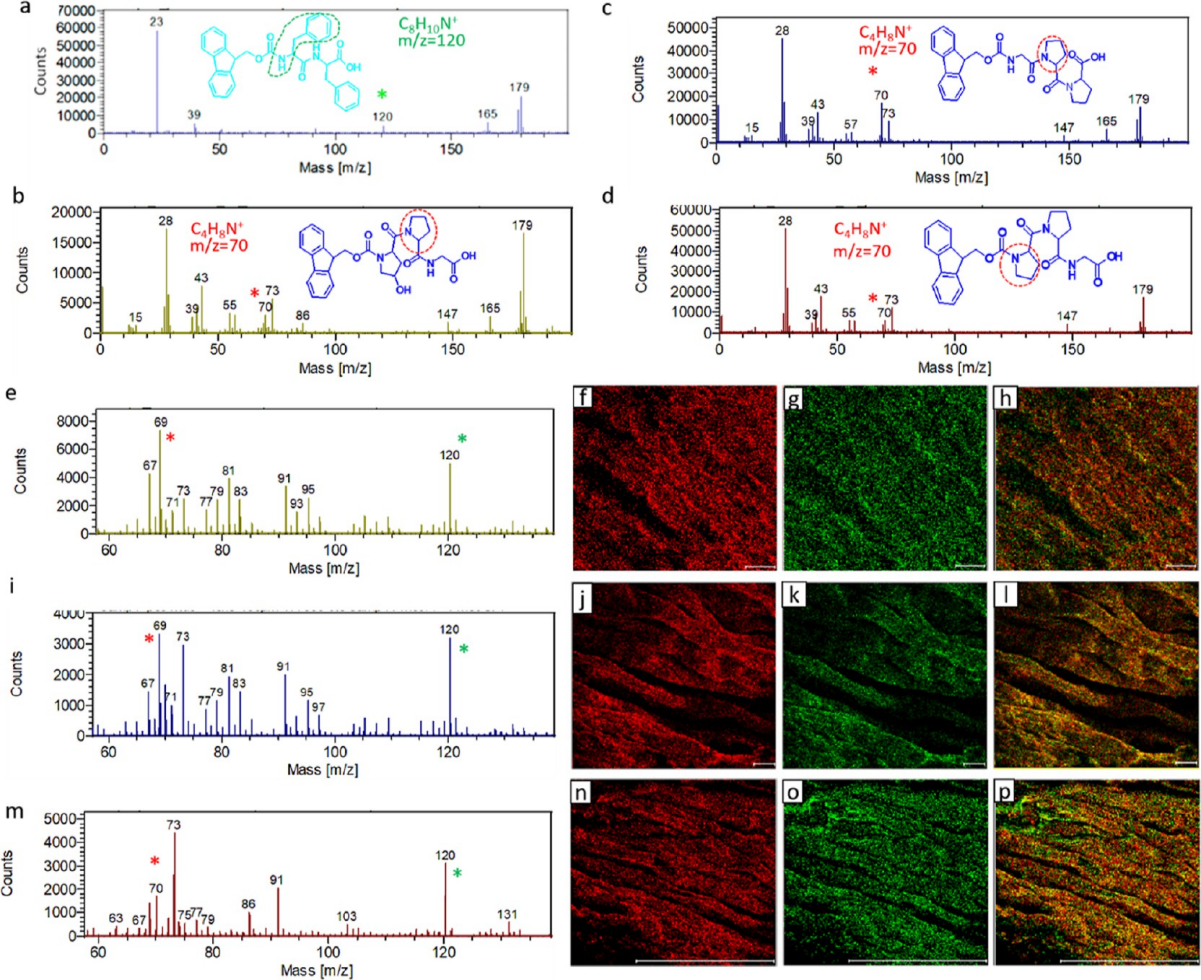
ToF-SIMS analysis of the chemical composition of the hybrid hydrogels. (a–d) ToF-SIMS mass spectra of the single peptides: (a) Fmoc–Phe–Phe, (b) Fmoc–Hyp–Pro–Gly, (c) Fmoc–Gly–Pro–Pro, and (d) Fmoc–Pro–Pro–Gly. (e–h) H2: Fmoc–Phe–Phe/Fmoc–Hyp–Pro–Gly. (i–l) H3: Fmoc–Phe–Phe/Fmoc–Gly–Pro–Pro. (m–p) H4: Fmoc–Phe–Phe/Fmoc–Pro–Pro–Gly. (e,i,m) Mass spectra. (f–h,j–l,n–p) ToF-SIMS ion images of (f,j,n) collagen-biomimetic tripeptide labeled in red, (g,k,o) peptide 5 labeled in green, and (h,l,p) overlapping image. Scale bars: (f–h,j–l) 10; (n–p) 100 μm.

To confirm that the nanostructures formed by supramolecular co-assembly were indeed composed of both Fmoc–Phe–Phe and the collagen-mimicking peptides, we used time-of-flight secondary ion mass spectrometry (ToF-SIMS) analysis for chemical identification.^37,50^ High-resolution mass spectrum in positive ion mode of pure Fmoc–Phe–Phe included an intense peak at *m*/*z* 120 corresponding to the C_8_H_10_N^+^ ion, while all the collagenous tripeptides exhibited a characteristic positive ion peak at *m*/*z* 70 corresponding to the C_4_H_8_N^+^ ion (Figure 4a–d). The presence of both ion peaks at 120 and 70 in the mass spectra of dried fibers from the hybrid systems confirm the presence of both components in the nanostructures, which is the first step to proving the co-assembly (Figure 4e,i,m). Next, chemical ion mapping was employed by defining the mass at 120 as green and the mass at 70 as red and evaluating their presence over a precise area of the fibers (Figure 4f–h,j–l,n–p). By overlapping the red and green mapping in a single image, a distinctive yellow color could be observed throughout the fibers reflecting the presence of both ions throughout indicative of the strong co-assembly in the hybrid systems (Figure 4h,i,p).

### Mechanical Rigidity of the Single Fibers

**Figure 5:**
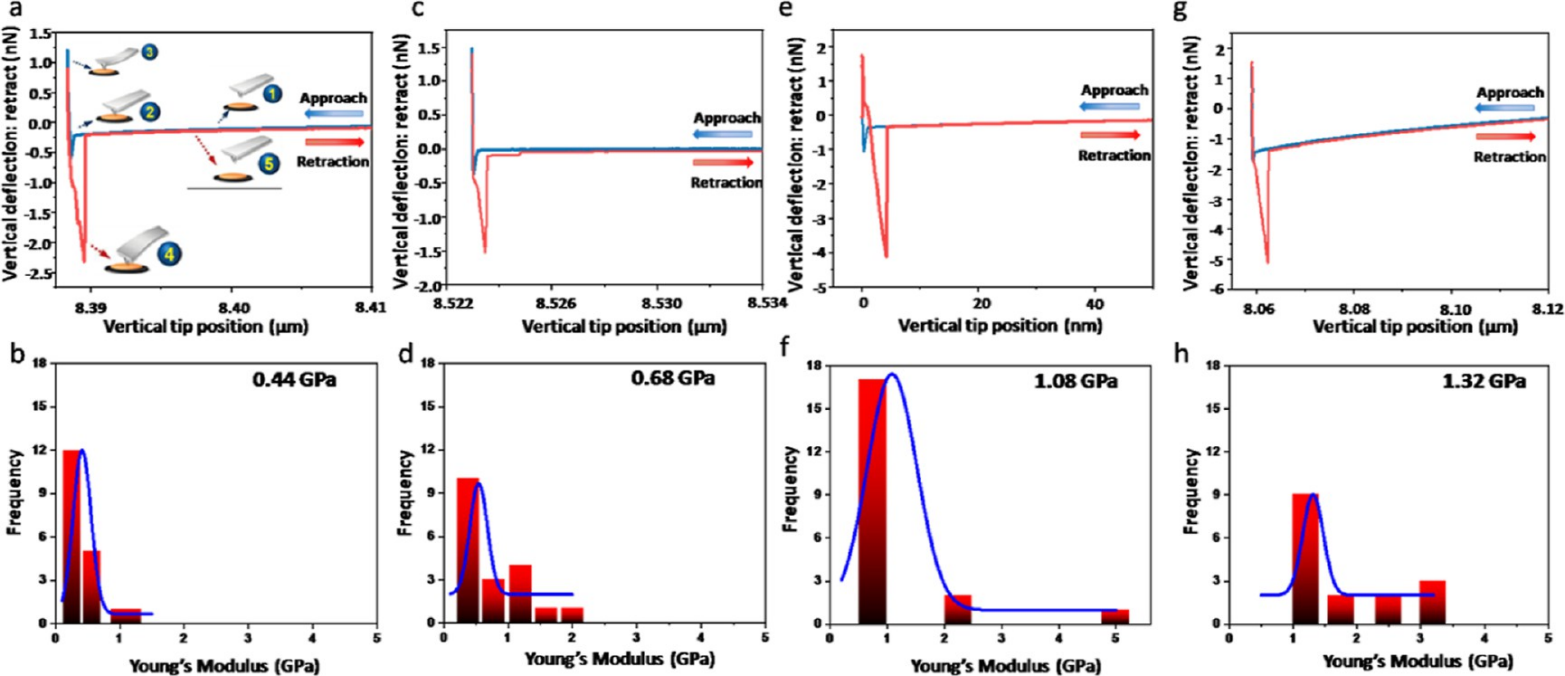
Mechanical properties of self-assembled nanostructures of the hybrid hydrogels. (a,c,e,g) Typical force–displacement curves and (b,d,f,h) statistical Young’s moduli distributions from ∼20 measurements each on (a,b) H1: Fmoc–Phe–Phe/Fmoc–Gly–Pro–Hyp, (c,d) H2: Fmoc–Phe–Phe/Fmoc–Hyp–Pro–Gly, (e,f) H3: Fmoc–Phe–Phe/Fmoc–Gly–Pro–Pro, and (g,h) H4: Fmoc–Phe–Phe/Fmoc–Pro–Pro–Gly.

To understand whether the significant differences in the rigidity of the four hybrid hydrogels are derived from differences at the single fiber level, we measured the mechanical properties of single fibers.^51,52^ For this purpose, we used an AFM-based nanoindentation method, which enables us to associate morphological information with local nanometer-scale mechanical properties.^53,54^ The AFM cantilever approached toward the surface of a single fiber on a flat mica sheet and retracted at a constant speed of 2 μm s^–1^ (Figure 5). This measurement yields a force *versus* distance curve, from which the mechanical properties, and specifically the Young’s modulus, can be quantified. The statistical Young’s modulus of single fibers could be obtained by analyzing a series of fibers after fitting the approaching traces using the Hertz model, yielding values of 0.44, 0.68, 1.08, and 1.32 GPa for H1–H4, respectively (Figure 5). Thus, the order of rigidity of the four hybrid fibers is H1 < H2 < H3 < H4, the same order as the corresponding storage modulus of the hydrogels.

These results indicate that co-assembly between Fmoc–Phe–Phe and the two tripeptides with adjacent proline moieties (Fmoc–Gly–Pro–Pro and Fmoc–Pro–Pro–Gly) produces a more mechanically stable assembly, again highlighting the impact of subtle sequence differences in directing the macroscopic properties of the hybrid hydrogels.

### Piezoelectric Properties of the Fmoc–Gly–Pro–Hyp Single Crystal

To further verify the collagen-like properties of the tripeptides, specifically, the presence of piezoelectricity, density functional theory (DFT) calculations were performed on the Fmoc–Gly–Pro–Hyp (peptide 1) single crystal. These predictions indicate a significant piezoelectric response in the minimal model tripeptide units that can potentially be exploited in future in the hybrid co-assemblies. The orthorhombic symmetry of the crystal allowed for three non-zero shear piezoelectric constants (Table 1). The supramolecular packing was such that no net dipole was present in the equilibrium unit cell, and a piezoelectric response could thus only be generated by applying a shear force along any crystallographic axis (Figure S11). The piezoelectric charge constants, measured in C/m^2^, were found to be low-to-average on the spectrum of piezoelectric biomolecular crystal responses.^55^ The predicted values of *e*_14_, *e*_25_, and *e*_36_ were 0.040, 0.010, and 0.003 C/m^2^, respectively. To calculate the piezoelectric strain constants *d*_*ij*_, we divided the charge tensor by the elastic stiffness tensor in the crystal (Table 2, which shows as expected far higher Young’s modulus than the experimental hydrogels). The maximum predicted strain constant was *d*_14_ with a value of 8.6 pC/N, similar to that of inorganic material zinc oxide (ZnO). The corresponding predicted *g*_14_ voltage constant of 266 mV m/N exceeds that of both lead zirconatetitanate (PZT) and polyvinylidene fluoride, illustrating the excellent efficiency of this ultra-short helical building motif, similar to that of collagen.^56,57^ Examination of the crystal supramolecular packing showed that the low elastic anisotropy was primarily due to the large Fmoc protecting group that stabilized the crystal along all three crystallographic axes. The tripeptide molecular backbone aligns parallel to the *a* axis, increasing the elastic stiffness in this direction by 25%. The moderate-to-low piezoelectric polarization was due to the multi-directional O–H···O HBs that formed between peptide molecules.

**Table 1:** Piezoelectric Charge, Strain, and Voltage Constants of Fmoc–Gly–Pro–Hyp Single Crystals as Predicted by DFT Calculations

piezoelectric constant, index *ij*	*e*_*ij*_ (C/m^2^)	*d*_*ij*_ (pC/N)	*g*_*ij*_ (mV m/N)
14	0.040	8.6	266
25	0.010	2.1	71
36	0.003	0.49	18

**Table 2:** Elastic Stiffness Tensor Diagonal Components of Fmoc–Gly–Pro–Hyp Single Crystals, as Predicted by DFT Calculations

elastic stiffness constant	Fmoc–Gly–Pro–Hyp (GPa)
*c*_11_	25.2
*c*_22_	19.6
*c*_33_	20.8
*c*_44_	4.67
*c*_55_	4.57
*c*_66_	6.95
Young’s modulus	12.1

### Supramolecular Assembly Characterization by MD Simulations

**Figure 6:**
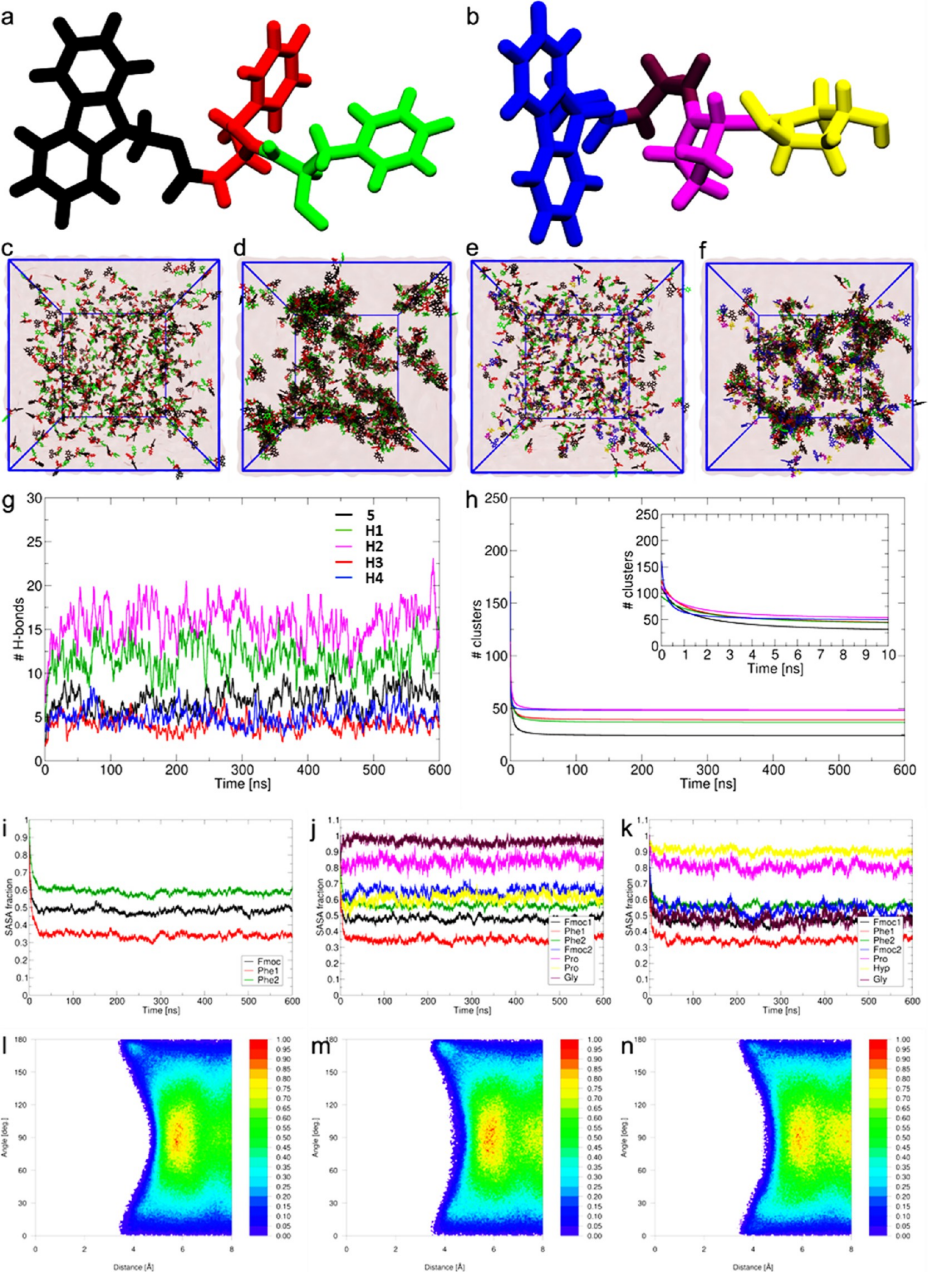
Supramolecular ordering of co-assemblies predicted from atomistic MD simulations. (a) Representation of the Fmoc–Phe–Phe monomer. Fmoc is colored black, the first Phe is red and the second Phe is green. (b) Representation of the Fmoc–Gly–Pro–Hyp monomer. Fmoc is colored blue, Gly brown, Pro magenta, and Hyp yellow. (c–f) Structures of (c,d) Fmoc–Phe–Phe self-assembly and (e,f) Fmoc–Phe–Phe/Fmoc–Gly–Pro–Hyp co-assembly. (c,e) Starting randomly dispersed structure of solvated molecules. (d,f) Final assembled structure after 600 ns of equilibrated dynamics in water at room temperature. Water is depicted by the transparent surface and peptides are depicted as sticks with the same color coding as in panels (a,b). (g) Running average of the number of HBs per peptide as a function of time within each self-/co-assembly. Fmoc–Phe–Phe, H1: Fmoc–Phe–Phe/Fmoc–Gly–Pro–Hyp, H2: Fmoc–Phe–Phe/Fmoc–Hyp–Pro–Gly, H3: Fmoc–Phe–Phe/Fmoc–Gly–Pro–Pro, and H4: Fmoc–Phe–Phe/Fmoc–Pro–Pro–Gly. (h) Number of peptide clusters as a function of time, with the same line coloring as used in panel (g). A second-order fit to the data is shown, with the raw data given in Figure S6. The inset shows the first 10 ns of data, highlighting the distinct kinetic and thermodynamic behaviors of each assembly. (i–k) Time evolution of the SASA fraction of different groups for (i) 5: Fmoc–Phe–Phe, (j) H4: Fmoc–Phe–Phe/Fmoc–Pro–Pro–Gly, and (k) H1: Fmoc–Phe–Phe/Fmoc–Gly–Pro–Hyp. (l–n) Computed free energy landscape for each assembly mapped over the last 100 ns of dynamics, highlighting the creation of π–π stacks: (l) Fmoc–Phe–Phe, (m) H4: Fmoc–Phe–Phe/Fmoc–Pro–Pro–Gly, and (n) H1: Fmoc–Phe–Phe/Fmoc–Gly–Pro–Hyp. The complete modeling data set with all properties calculated for all the co-assemblies is presented in the Supporting Information.

Finally, to map the supramolecular assembly that guides the formation of hydrogel scaffolds at the atomic scale, we performed MD simulations of the self-assembly of pure Fmoc–Phe–Phe, and the H1–H4 co-assemblies, starting from free molecules placed randomly in large water boxes. Representative molecular structures of Fmoc–Phe–Phe and Fmoc–Gly–Pro–Hyp monomers are shown in Figure 6a,b, respectively. For each assembly, 400 peptide molecules were randomly placed in a large cubic box with an edge length of 17 nm with an encompassing medium of approximately 72 000 water molecules and 2000 DMSO molecules. This high concentration of peptides was used to increase the rate of assembly, allowing us to observe the initial stages of hydrogel formation at full atomic resolution within several hundred nanoseconds of dynamics (Figure 6). The molar ratio of Fmoc–Phe–Phe/Fmoc–tripeptide was maintained at 2:1 to match the experimental conditions used in the co-assembly of the hydrogels.

The computed final structures indicated that starting from randomly dispersed states (Figures 6c,e and S12b,d,f), the pure Fmoc–Phe–Phe and all four hybrid systems aggregated into large and relatively compact clusters within 600 ns (Figures 6d,f and S12c,e,g). Comparison of the self-assembly of Fmoc–Phe–Phe (Figure 6d and Movie S1) and the co-assembly of Fmoc–Phe–Phe/Fmoc–Gly–Pro–Hyp (Figure 6f and Movie S2) highlighted the tendency of the Fmoc–Phe–Phe/Fmoc–Gly–Pro–Hyp combination to very rapidly form large, multi-molecule superstructures similar to Fmoc–Phe–Phe, consistent with the experimental kinetic data (Figure 2). To account for the contribution of HBs in the assembly of the molecules into hydrogel scaffolds, we computed the numbers and distributions of HBs (Figures S13 and S14 and Supporting Information Note S1.2) in the assembled superstructures. The running averages of the number of HBs as a function of time (Figure 6g) showed that their contributions in the co-assembly of Fmoc–Phe–Phe/Fmoc–Hyp–Pro–Gly (H2) is higher than in Fmoc–Phe–Phe/Fmoc–Gly–Pro–Hyp (H1), suggesting that the participation of the terminal Hyp group in forming inter-peptide HBs is restricted by its hydrogen bonding with water. On the other hand, the presence of a Pro residue in place of Hyp at position 1 or 3 of the tripeptides [in the case of Fmoc–Phe–Phe/Fmoc–Gly–Pro–Pro (H3) and Fmoc–Phe–Phe/Fmoc–Pro–Pro–Gly (H4)] significantly reduced their capacity to form supramolecular scaffolds *via* hydrogen bonding. The controlled, rigid self-assembly of pure Fmoc–Phe–Phe (5) displayed as expected a similarly lower affinity of stabilization through HBs, due to preferred engagement in π–π stacking.

We monitored the number of peptide clusters formed as a function of simulation time (Figure S12a) to identify the rate of formation and cohesive strength of the supramolecular peptide self-assembly and co-assemblies. Further details of clustering analyses are provided in Supporting Information Note S1.1. Figure 6h shows a second-order fit of the plot as a function of time. The pure Fmoc–Phe–Phe (5) self-assembly proceeded *via* an initial rapid drop in the number of clusters to ∼25, indicating the strongest propensity to form large supramolecular clusters, and remained in this saturated state for 600 ns. The Fmoc–Phe–Phe/Fmoc–Gly–Pro–Hyp (H1) and Fmoc–Phe–Phe/Fmoc–Gly–Pro–Pro (H3) co-assemblies were stabilized with ∼37 and ∼39 clusters, respectively. Fmoc–Phe–Phe/Fmoc–Hyp–Pro–Gly (H2) and Fmoc–Phe–Phe/Fmoc–Pro–Pro–Gly (H4) underwent a slower assembly process and remained at the steady state of ∼50 clusters until the end of the simulation. Our models confirm the experimental indications that the assemblies are both kinetically and thermodynamically controlled.

To complement the clustering analyses, the time evolution of the fraction of solvent accessible surface area (SASA) of individual molecules was measured relative to their initial randomly dispersed states. The data (Figures 6i–k and S15a,b and Supporting Information Note S1.3) suggested that Fmoc–Phe–Phe underwent a more rapid, significant, and sustained burial of peptide units in water (Figure 6i) and predicted that the formation of Fmoc–Phe–Phe/Fmoc–Gly–Pro–Hyp superstructures could be the fastest (Figure 6k) among the co-assembling peptides as the fraction SASA losses of their Fmoc–Gly fragment were equivalent to that of the Fmoc–Phe–Phe fragment. By contrast, in the co-assembly of Fmoc–Phe–Phe/Fmoc–Pro–Pro–Gly (Figure 6j), the Fmoc–Pro portion was relatively more solvent-exposed (see Supporting Information Note S1.3) than Fmoc–Phe–Phe, similar to the other co-assembling peptide groups (see Figure S15a,b). The simulations show how co-assembly of Fmoc–Phe–Phe/Fmoc–Gly–Pro–Hyp is driven by expedited burial of the Fmoc–Phe–Phe groups.

To understand the role of π–π interactions in driving the assembly process, the free energy landscapes (FEL) of intermolecular π–π stacking were plotted as a function of the centroid to centroid distance between rings on neighboring molecules (within a threshold of 8 Å) and the angle between the normal vectors of the pairs (Figures 6l–n and S15e,f). The FEL of the Fmoc–Phe–Phe self-assembly revealed primarily T-shaped π–π stacking configuration (∼90°) at a centroid–centroid distance of ∼5–6 Å (red basin). Some π–π sandwich stacking (cyan basin) or parallel displaced stacking (180°) was observed at shorter distances of ∼4 Å (Figure 6l). Figure S15c shows a representative supramolecular assembly of Fmoc–Phe–Phe with the prominent π–π network colored in yellow. The π–π network in the co-assemblies of Fmoc–Phe–Phe and the two Fmoc–tripeptides containing adjacent proline residues (Fmoc–Phe–Phe/Fmoc–Gly–Pro–Pro and Fmoc–Phe–Phe/Fmoc–Pro–Pro–Gly) showed higher resemblance to the very rigid pure Fmoc–Phe–Phe structure that produced a strong and tightly packed peptide supramolecular organization (Figures 6m and S15e). By contrast, the model showed minimal π–π parallel stacking in the case of the Fmoc–Phe–Phe/Fmoc–Gly–Pro–Hyp co-assembly (Figure 6n), with a small population of T-shaped stacking of ∼5–6 Å and an additional small minimum at longer distances (around 8 Å and 90°) of possibly laterally displaced T-shaped stacking. Figure S9d shows a representative supramolecular co-assembly of Fmoc–Phe–Phe/Fmoc–Gly–Pro–Hyp with the weak π–π network colored in yellow. The computed extents of π–π networking in the assemblies (in Figure S15c,d) reflect the computed sizes of the supramolecular clusters (Figure 6h), with the models showing that burial of the highly interwoven Fmoc–Phe–Phe π–π network drives the Fmoc–Phe–Phe/Fmoc–Gly–Pro–Hyp co-assembly (see Movies S1 and S2). By contrast, the higher incidence of co-peptide HBs (discussed above) coupled with reduced π–π stacking (see details in Supporting Information Note S1.4) could explain the rapid formation of the weak superstructure of Fmoc–Phe–Phe with the Hyp-containing peptide, Fmoc–Gly–Pro–Hyp (H1).

Finally, comparing the MD results with the measured Young’s moduli (Figure 5) confirms that the co-assembly of Fmoc–Phe–Phe and the two tripeptides containing adjacent proline moieties (Fmoc–Phe–Phe/Fmoc–Gly–Pro–Pro and Fmoc–Phe–Phe/Fmoc–Pro–Pro–Gly) produces a stronger and more tightly packed supramolecular organization mainly through π–π stacking. The increased hydrogen bonding capacity of the Hyp-containing peptides (Fmoc–Phe–Phe/Fmoc–Gly–Pro–Hyp and Fmoc–Phe–Phe/Fmoc–Hyp–Pro–Gly) accelerates assembly but results in more flexible, more open, water-rich assemblies (Figure S16).

## Conclusions

In this report, we present a detailed study of the structural design criteria governing the formation of a collagen-helix backbone by a minimal single triplet motif in a series of engineered peptides. The formation of a polyproline II helical structure by all four tripeptides demonstrates that the helical orientation of the Gly–Xaa–Yaa triplet is generic in nature and is not dependent on side-chain modification, sequence order, or the presence of Hyp. Co-assembly of these designed collagen-mimetic peptides with a non-collagenous hydrogelator produces mechanically rigid hydrogels with collagenous traits. The robustness of the co-assembled hydrogels varied significantly in different hybrid systems according to the degree and type of supramolecular interactions facilitated by proper positioning of the amino acids in the sequence of the shortest collagenous motifs. Collagen plays a critical role in tissue structure, repair, and regeneration, yet due to the difficulty to extract and use it in its natural state, obtaining true collagen-like designed materials represents a major challenge for a wide variety of tissue engineering and broader nano-/bioapplications. Our results present a proof of concept for the design principle of selecting the shortest possible collagen-like peptide motifs for co-assembly into hybrid systems with customized properties, potentially providing functional biomaterials for future regenerative medicine applications.

## Supporting Information Available

SEM, absorbance kinetics measurement of the gels, ToF-SIMS analysis of the fibers, AFM for the study of mechanical properties of fibers, DFT calculations, details of MD simulation setup, supramolecular clustering analyses, contribution from inter-peptide HBs, role of encapsulated water in directing hydrophobic and hydrophilic inter-peptide contacts, free energy mapping of buried π–π networks, crystal structure of Fmoc–Gly–Pro–Hyp, concentration-dependent spectra, gelation study of the four tripeptides, stability of the hybrid hydrogels, gelation kinetics, time sweep oscillatory measurements, dynamic strain sweep experiments, recovery of mechanical properties of gels, characterization of native collagen, formation of peptide clusters as a function of time, running average of the number of HBs, distribution of the number of HBs, timeline of the SASA fraction, and schematic of the self-assembly mechanism of hybrid systems (PDF)
Self-assembly of Fmoc–Phe–Phe (MP4)
Co-assembly of Fmoc–Phe–Phe/Fmoc–Gly–Pro–Hyp (MP4)
