# Supplementary material for: Molecular Engineering of Rigid Hydrogels Co-assembled from Collagenous Helical Peptides Based on a Single Triplet Motif
Source: ACS Appl Mater Interfaces. 2022 Oct 7;14(41):46827–40. doi: 10.1021/acsami.2c09982 (PMC9585512; doi:10.1021/acsami.2c09982)
Supplement: Supplementary file 1 — am2c09982_si_001.pdf [file am2c09982_si_001.pdf]

Supporting Information

Molecular Engineering of Rigid Hydrogels Co-Assembled from Collagenous Helical Peptides Based on Single Triplet Motif

Santu Bera,[†] *Pierre-Andre Cazade,*[‡] *Shayon Bhattacharya,*[‡] *Sarah Guerin,*[‡] *Moumita Ghosh,*[†,‖] *Francesca Netti,*[†] *Damien Thompson,*[‡] *and Lihi Adler-Abramovich*[†]*

[†]Department of Oral Biology, The Goldschleger School of Dental Medicine, Sackler Faculty of Medicine, The Center for Nanoscience and Nanotechnology, and The Center for the Physics and Chemistry of Living Systems, Tel Aviv University, Tel Aviv 6997801, Israel.
Email: lihia@tauex.tau.ac.il

[‡]Department of Physics, Bernal Institute, University of Limerick, V94T9PX, Ireland.

METHODS

Scanning Electron Microscopy (SEM). A 5 μL aliquot was allowed to dry on a microscope glass cover slip under ambient conditions overnight, and then coated with Au. SEM images were recorded using a JSM-6700F FE-SEM (JEOL, Tokyo, Japan) operating at 20 kV.

Absorbance Kinetics Measurement of the Gels. Samples of 150 μL of each hydrogel were placed in wells of a 96-well plate. Absorbance at 350 nm was measured every 3 min for 2 h using a TECAN Infinite M200PRO plate reader.

Time-of-Flight Secondary Ion Mass Spectrometry (ToF-SIMS). A bundle of thin fibrils of hybrid hydrogels was prepared between two thin capillaries by drying the gels. The dried fibril was deposited on a silicon wafer and analyzed by a PHI model 2100 TRIFT II ToF-SIMS instrument. The system useda pulsed primary ion beam to desorb and ionize species from the amino acids' surface. The resulting secondary ions were accelerated into a mass spectrometer, where they were mass analyzed by measuring their time-of-flight from the sample surface to the detector. In addition, an image was generated by rostering a finely focused beam across the sample surface. Due to the parallel detection nature of ToF-SIMS, the entire mass spectrum was acquired from every pixel in the image. The ions related to 120 and 70 m/z were used to identify and evaluate the ionic image of the co-assembled hybrid hydrogels. The mass spectrum and the secondary ion images were then used to determine the composition and distribution of sample surface constituents.

Atomic Force Microscopy. AFM images were obtained by depositing 5 μL of the hydrogel solution onto freshly cleaved V1 grade mica (Ted Pella) immediately after preparation. The samples were allowed to dry under ambient conditions for 24 h. The samples were imaged using

AFM (JPK Instruments AG) performed using Nano Wizard 3 with 5 N/m spring constant tips and a resonance frequency of ~150 kHz in soft tapping mode. The images were processed and analyzed by the JPK Data Processing software. The AFM nanoindentation experiments were performed using the same AFM. The force curves were obtained using the commercial software from JPK and analyzed by a custom-written procedure based on Igor Pro 6.12 (Wavemetrics Inc.). Silicon nitride cantilevers (Budget Sensors Company with the triangle shape, length 100 µm, width 16 µm, thickness 520 nm, frequency in air: ~30 kHz) were used in all experiments. In a typical experiment, the QI mode was used to detectthe Young’s modulus (conditions: pixels: 126 × 126; Z length: 0.3 µm; extend and retract speed: 2µm s^{-1}, Z resolution: 80 000 Hz). The spring constant of the cantilevers was 0.27 N m^{-1}, and the maximum loading force was set at 1.34 nN. All AFM experiments were carried out in air at room temperature. In a typical measurement, the cantilever was brought above the crystals with the help of an optical microscope. Then, the cantilever was extended to the surface of the crystal and retracted at a constant speed of 2µm s^{-1}. The extending and retracting force curves were recorded. The Young’s modulus values of the crystals were obtained by fitting the extend curve to the Hertz model.

Density Functional Theory Calculations. Electromechanical properties were predicted from periodic DFT calculations[1] on the single crystal using the VASP[2] code. Electronic structures were calculated using the PBE functional[3] with Grimme-D3 dispersion corrections[4] and projector augmented wave (PAW) pseudopotentials[5]. The crystal structure was optimised using a plane wave cut-off of 600 eV with a 4x4x4 k-point grid. A finite differences method was used to calculate the stiffness tensor, with each atom being displaced in each direction by ± 0.01 Å, and piezoelectric strain constants and dielectric tensors calculated using Density Functional

Perturbation Theory[6] (DFPT), with a plane wave cut-off of 600 eV and k-point sampling of 2x2x2. Young's Moduli were derived from the stiffness and its inverse compliance matrix components. Values are presented as Voigt-Reuss-Hill averages[7,8]. Crystal structures were visualised using VESTA[9].

Details of MD Simulation Setup

400 peptide molecules were placed in a cubic 17 nm X 17 nm X 17 nm box containing 2000 DMSO molecules. The Fmocresidue was parametrized with ParamChem[10,11], which provides the CHARMM General Force Field (CGenFF)[12] parameters. Additionally, the partial charges for Fmoc atoms were derived using the Restrained Electrostatic Potential (RESP)[13] scheme based on quantum mechanical calculations with Gaussian[14], followed by charge fitting with Antechamber[15]. The peptide molecules and DMSO were represented by the CHARMM 36m[16] force field, and solvated with 72,000 molecules of CHARMM-modified TIP3P[16] explicit water.

MD simulations were carried out using the Gromacs 2018.4[17] package with a time step of 2 fs using the Leap frog integrator.[18] Bond lengths to hydrogen were constrained using the LINCS[19] (protein) and the SETTLE[20] (water) algorithms. Background ions were added to neutralise full protein formal charges. Long-range electrostatics were treated by the Particle mesh Ewald (PME) method.[21] Protein and non-protein molecules (water, DMSO and ions) were coupled separately to an external heat bath (300 K) with acoupling time constant of 1 ps using the velocity rescaling method.[22] All systems were minimised for 100 ps, and equilibrated for 500 ps in constant volume NVT ensemble followed by another 500ps of NPT equilibration with the reference pressure set at 1 bar and a time constant of 5ps using the Parrinello-Rahman barostat.[23] The production runs were carried out for 600 ns in constant pressure NPT ensemble. Structures

were saved every 20 ps. MD trajectories of the Fmoc-Phe-Phe assembly and Fmoc-Phe-Phe/Fmoc-Gly-Pro-Hypco-assembly are provided in movies S1 and S2.

SUPPLEMENTARY NOTES

S1. Computed Properties from MD Simulations

S1.1 Supramolecular Clustering Analyses. To compute the formation of self-assembling and co-assembling molecular clusters as a function of time, we used the *gmxclustsize* routine in Gromacsat a molecule-molecule cut-off distance of 0.5 nm. Figure S12 shows the number of peptide clusters formed as a function of simulation time. The pure self-assembling Fmoc-Phe-Phe and the four co-assembling Fmoc-Phe-Phe/Fmoc-tripeptides revealed the formation of peptide clusters evident from a rapid drop in the number of clusters (#clusters) within the first 10 ns of the simulation (inset, Figure S12 and main text Figure 6). As expected, pure Fmoc-Phe-Phe formed the largest clustersas the Phe-Phe units formed tight supramolecular networks *via* π−π extensive packing. The rank order of clusters formation amongst the co-assemblies was Fmoc-Phe-Phe/Fmoc-Gly-Pro-Hyp>Fmoc-Phe-Phe/Fmoc-Gly-Pro-Pro>Fmoc-Phe-Phe/Pro-Pro-Gly≈Fmoc-Phe-Phe/Hyp-Pro-Gly. The superstructuring of pure Fmoc-Phe-Phe and of the fastest co-assembly system Fmoc-Phe-Phe/Fmoc-Gly-Pro-Hyp is shown in the main text Figure 6c-f. The superstructures formed for other co-assembled systems are shown in Figure S12b-g.

S1.2 Contribution from Inter-Peptide Hydrogen Bonds (HBs). The number of HBs formed during assembly was calculated with a characteristic donor-acceptor distance of 0.3 nm and an angle of 20° using VMD software.[24]Figure S13 shows the number of HBs (#HBs) as a function of simulation time. For comparison purposes, we again included the total #HBs between all

peptide molecules in the systems (Figure S13a) as in Figure 6g, together with the normalized #HBs between Fmoc-Phe-Phe molecules only (Figure S13b) in its self-assembly and in the co-assembly with the tripeptides, the normalized #HBs only between co-peptide molecules and their comparison with Fmoc-Phe-Phe (Figure S13c), and the #HBs between Fmoc-Phe-Phe and each co-peptide (Figure S13d). Although similar #HBs were formed between Fmoc-Phe-Phe molecules (Figure S13b) in their co-peptide systems, a slightly higher number was sampled during self-assembly of pure Fmoc-Phe-Phe. In the co-assembly of Fmoc-Phe-Phe and the tripeptides (Figure S13d), some Fmoc-Phe-Phe units formed HBs withthe tripeptides, and removal of the Hyp hydroxyl group (in Fmoc-Phe-Phe/Fmoc-Gly-Pro-Pro and Fmoc-Phe-Phe/Fmoc-Pro-Pro-Gly) reduces the probability of forming co-assembled HBs. A similar trend was observed with regards to the self-assembling tripeptide HBs (Figure S13c), where the Hyp residue increased the HB population in Fmoc-Phe-Phe/Fmoc-Gly-Pro-Hyp and Fmoc-Phe-Phe/Fmoc-Hyp-Pro-Gly.

We also mapped the % distribution of #HBs, which showed a broader distribution of the higher number of total HBs for Fmoc-Phe-Phe/Fmoc-Gly-Pro-Hyp and Fmoc-Phe-Phe/Fmoc-Hyp-Pro-Glyand anarrower distribution of the lower #HBs for Fmoc-Phe-Phe/Fmoc-Gly-Pro-Pro and Fmoc-Phe-Phe/Fmoc-Pro-Pro-Gly (Figure S14a). The % densities of self-assembling Fmoc-Phe-Phe HBs were very similar to each other with occasional sharper peaks for Fmoc-Phe-Phe/Fmoc-Pro-Pro-Gly(Figure S14b). A high density of low #HBs was observed for Fmoc-Phe-Phe/Fmoc-Gly-Pro-Pro and Fmoc-Phe-Phe/Fmoc-Pro-Pro-Gly, reflecting as expected both reduced tripeptide-tripeptide HB contacts (co-peptides; see Figure S14c) and reduced co-assembling HBs with Fmoc-Phe-Phe (Figure S14d), accounted for the substitution of Hyp for Pro.

S1.3 Role of Encapsulated Water in Directing Hydrophobic and Hydrophilic Inter-Peptide Contacts. In order to identify the different roles of the individual groups in the Fmoc-Phe-Phe self-assembly and the four co-assemblies in the formation of the hydrogel scaffolds, we computed the fraction of their solvent accessible surface area (SASA) relative to their initial randomly dispersed states (Figure 6i-k and S15a, b). The SASA fractions of Fmoc-Phe1-Phe2 assembly (where Phe1 is the first phenyl group and Phe2 is the second phenyl group in the sequence; a similar nomenclature is followed throughout for the residues in the tripeptidesaccording to their position in the sequence for direct comparison) rapidly droped during the first 25 ns for each group, in the order of Phe1 >Fmoc> Phe2. The gels remained in these super-structured states for the remainder of 600 ns MD (see Figure 6i). The mid-peptide Phe1 underwent maximum SASA loss indicating the potential to be buried inside the hydrophobic core of the aggregates with a propensity to engage in π-π T-stacks. The terminal Phe2 remained significantly more exposed to water, with the Fmoc unit intermediate relative to the two Phe units. After 600 ns, the SASA fraction values were 0.48 for Fmoc, 0.32 for Phe1 and 0.57 for Phe2 (see Figure 6i).

The SASA fractions of individual groups belonging to the Fmoc-Phe-Phe/Fmoc-Gly-Pro-Pro (Fmoc1-Phe1-Phe2/Fmoc2-Gly-Pro1-Pro2) co-assembly droped rapidly except for Pro2 (Figure S15a), which mostly remained exposed to the solvent (0.93 after600 ns). This is due to the charged C-terminus and “tail” position of this unit. The degree of burial was Phe1 > Fmoc1 > Phe2 ≈ Fmoc2 > Pro1 ≈ Gly> Pro2. The final SASA fraction values of 0.49 for Fmoc1, 0.37 for Phe1 and 0.59 for Phe2 suggest that Fmoc1-Phe1-Phe2 remained as buried during co-assembly as it did during the single self-assembly of Fmoc-Phe1-Phe2. In the co-assembling Fmoc2-Gly-Pro1-Pro2 peptide, Fmoc2 remained buried to a similar degree as Phe2 with a final SASA

fraction value of 0.65, followed by Pro1 and Gly (~0.75 each). Pro1 and Gly share similar SASA values as the latter is very small and does not exhibit any site for H-bonding or π-π stacking. Therefore, its exposure to the solvent is solely driven by Pro1. Similar rapid loss of SASA fractions was observed for theFmoc1-Phe1-Phe2 groups (Phe1 > Fmoc1 > Phe2) as opposed to the Fmoc2-Hyp-Pro-Gly groups in the co-assembly of Fmoc1-Phe1-Phe2/Fmoc2-Hyp-Pro-Gly (Figure S15b). Gly, the terminal group of the tripeptide, remained mostly exposed to the solvent (as opposed to Gly in Fmoc2-Gly-Pro1-Pro2), sometimes displaying a larger fraction (>1) than its initial randomly dispersed state. Pro as the penultimate group in Fmoc2-Hyp-Pro-Gly remained buried to a similar extent (0.8) as Pro1 in Fmoc2-Gly-Pro1-Pro2. Hyp following Fmoc remained more exposed (0.85) than Pro following Hyp. The order of the degree of SASA loss for the second tripeptide only was Fmoc2 > Pro >Hyp>Gly.

Rapid, significant, and sustained loss of SASA fractions was also observed for individual groups belonging to Fmoc1-Phe1-Phe2 (converged values of 0.35 for Phe1, 0.48 for Fmoc1 and 0.57 for Phe2) in the Fmoc1-Phe1-Phe2/Fmoc2-Pro1-Pro2-Gly (Fmoc-Phe-Phe/Fmoc-Pro-Pro-Gly) co-assembly (see Figure 6j) compared to groups in Fmoc2-Pro1-Pro2-Gly (0.6 for Pro2, 0.65 for Fmoc2, 0.85 for Pro1 and 0.95 for Gly). The terminal solvent-exposed Gly group transiently showed fractions larger than 1, like Fmoc2-Hyp-Pro-Gly (Figure S15b). The placement of Pro2 in the second position of the tripeptide as opposed to the terminal position in Fmoc2-Gly-Pro1-Pro2 significantly improved its participation in the assembly by burying in the core. As expected, SASA fractions of individual groups belonging to buried Phe1 were the lowest, as seen in all other cases in the co-assembly of Fmoc1-Phe1-Phe2/Fmoc2-Gly-Pro-Hyp. However, interestingly, the fraction SASA losses of the Fmoc-Gly portion of Fmoc2-Gly-Pro-Hyp were almost at par with Fmoc1-Phe1-Phe2 (order Phe2 >Gly ≈ Fmoc1 > Fmoc2 ≈ Phe1), in

contrast to the other co-assemblies (see Figure 6k). The MD data indicates that the rate of formation of Fmoc-Phe-Phe/Fmoc-Gly-Pro-Hyp superstructures may be the fastest amongst the co-assemblies, consistent with the measured kinetic data (Figure 2). The simulations also predict that replacing Pro with Hyp at the third position of the tripeptide sequences improves their ability to formwater-mediated hydrogen bonds in the hydrogel scaffolds (see Figure S13a and S13b for comparison).

S1.4 Free Energy Mappingof Buried π-π Networks. The free energy landscapes (FEL) of intermolecular π-π stacking were plotted as a function of the centroid distance between rings on neighboring molecules and the angle between the normal vectors of the pairs of Fmoc and/or Phe rings for Fmoc-Phe-Pheand pairs of Fmoc, Phe, Pro and, where present,Hyprings for Fmoc-Phe-Phe/Fmoc-Gly-Pro-Pro, Fmoc-Phe-Phe/Fmoc-Gly-Pro-Hyp, Fmoc-Phe-Phe/Fmoc-Pro-Pro-Gly, and Fmoc-Phe-Phe/Fmoc-Gly-Pro-Hyp within a threshold of 8 Å. These plots were generated by sampling the FEL during the last 100 ns of the simulation (500–600 ns). The FEL of Fmoc-Phe-Phe self-assembly was majorly T-shaped π-π stacking (planes of rings were perpendicular to each other) at a distance of ~5–6 Å (see Figure 6l and Figure S15c). There was also evidence of π-π parallelstacking or parallel displaced stackingat shorter distances of ~4 Å.

The FEL for the co-assemblies of the Fmoc-Gly-Pro-Pro, Fmoc-Hyp-Pro-Gly and Fmoc-Pro-Pro-Gly tripeptides with Fmoc-Phe-Phe (Figure S15e, f and 6m) again revealed primarily T-shaped π-π stacking configurations (at or around 90 degrees) at a centroid-centroid distance of ~5–6 Å (red basin) with less prominent (cyan basin) π-π sandwich or parallel displaced stacking (180 degrees) at ~4 Å. The minimal basin for T-shaped stacking (Figure S15d) of the Fmoc-Phe-Phe/Fmoc-Gly-Pro-Hyp system occured around 5–6 Å with an additional small minimum at longer distances of~8 Å (see Figure 6n). The maps show how both the local and long-range

structure of the stacking network depend critically on the sequence of the peptides. These results show the resemblance between the π−π network in the co-assemblies between Fmoc-Phe-Phe and the two tripeptides with adjacent proline moieties and the very rigid pure Fmoc-Phe-Phe self-assembled structure, which produces a tightly packed peptide supramolecular organization, as reflected in the measured mechanical strengths (Figure 2).

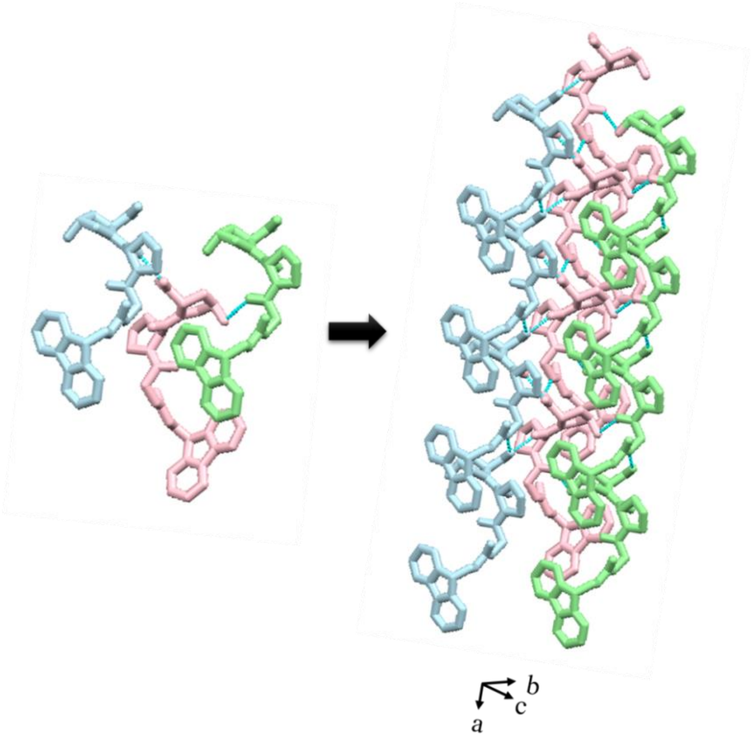

Figure S1 Crystal structure of Fmoc-Gly-Pro-Hyp (1).Side-by-side H-bond connection of a single helical chain with two nearby helical chains. For visualization, the three helices are colored differently. Intermolecular H-bonds are represented by blue dotted lines.

Table S1: Comparison of torsion angles for previously reported N-terminal Gly contacting hydrophobic tripeptides and current peptide.

Structure	Tripeptide	*Φ2*	*Ψ2*	Reference
Non-helical	L-Ala-Gly-Gly	-83.1	169.4	
	L-Leu-L-Pro-Gly	-66.7	160.6	
	Gly-L-Leu-L-Tyr	-131	148	
α-helix	Gly-L-Ala-L-Phe	-71.2	-33.4	Parthasarthy *et al.*
	Gly-Gly-L-Val	-77.0	-22.2	

	Gly-L-Ala-L-Val	-68.2	-38.3	
	Gly-L-Ala-L-Leu	-67.6	-39.3	
Polyproline II	Fmoc-Gly-Pro-Hyp	-66.2	150.3	Current study

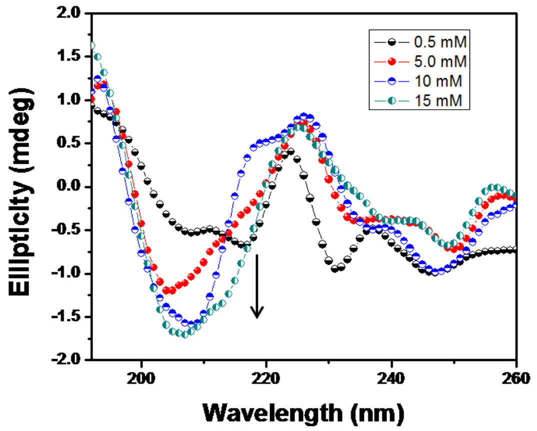

Figure S2 Concentration-dependent CD spectra of Fmoc-Hyp-Pro-Gly.

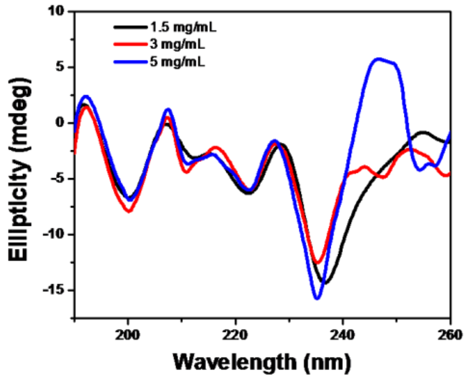

Figure S3 Concentration-dependent CD spectra of Fmoc-Leu-Pro-Hyp.

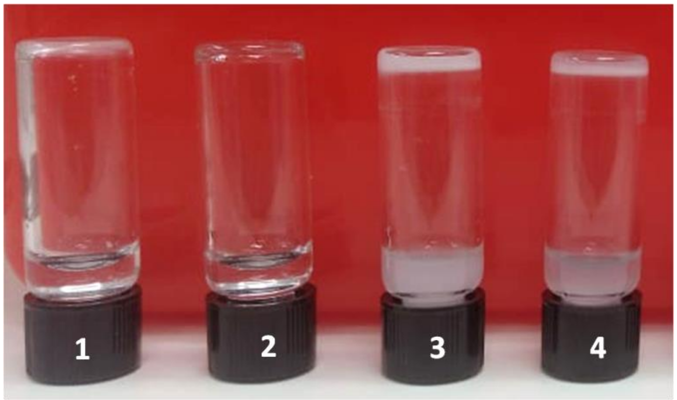

Figure S4 Gelation study of four tripeptides through solvent switch methods at a concentration of 5 mg/mL.

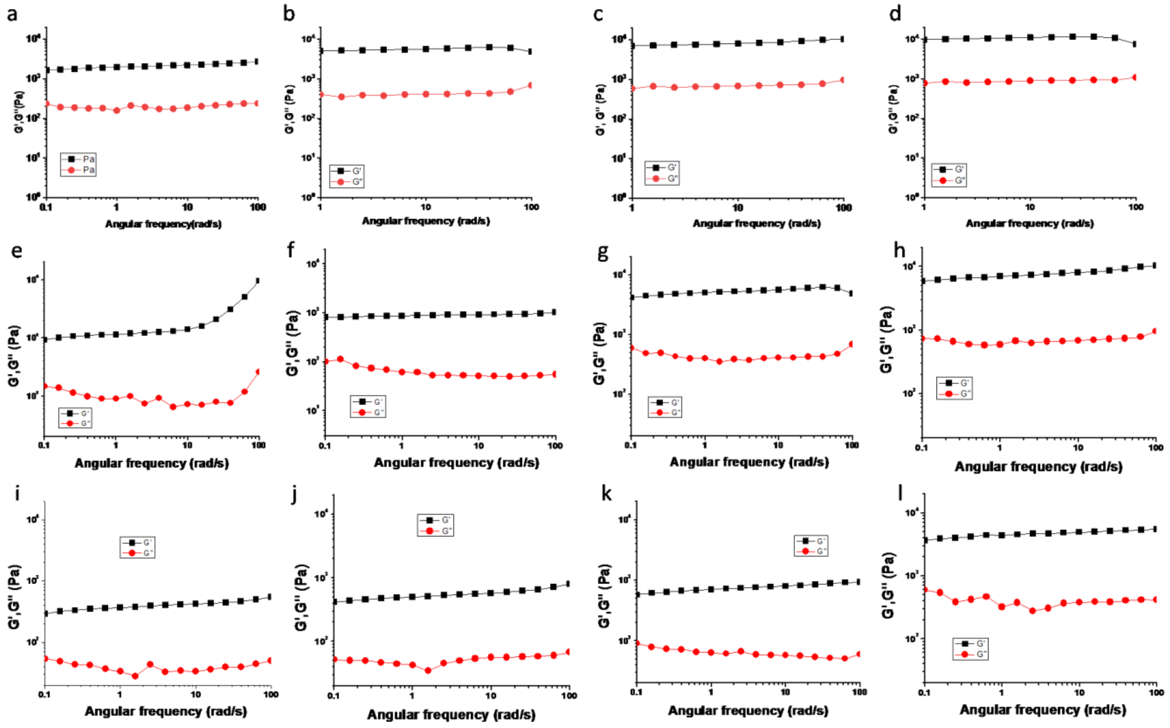

Figure S5 Stability of the hybrid hydrogels measured by rheological frequency sweep analysis. (a-l) Rheological frequency sweep analyies showing the storage modulus (G') and loss modulus (G"). (a, e, i) Fmoc-Phe-Phe:Fmoc-Gly-Pro-Hyp- 2:1, 1:1 and 1:2, respectively, (b, f, j) Fmoc-Phe-Phe/Fmoc-Hyp-Pro-Gly- 2:1, 1:1 and 1:2, respectively, (c, g, k) Fmoc-Phe-Phe/Fmoc-Gly-Pro-Pro- 2:1, 1:1 and 1:2, respectively, and (d, h, l) Fmoc-Phe-Phe/Fmoc-Pro-Pro-Gly- 2:1, 1:1 and 1:2, respectively.

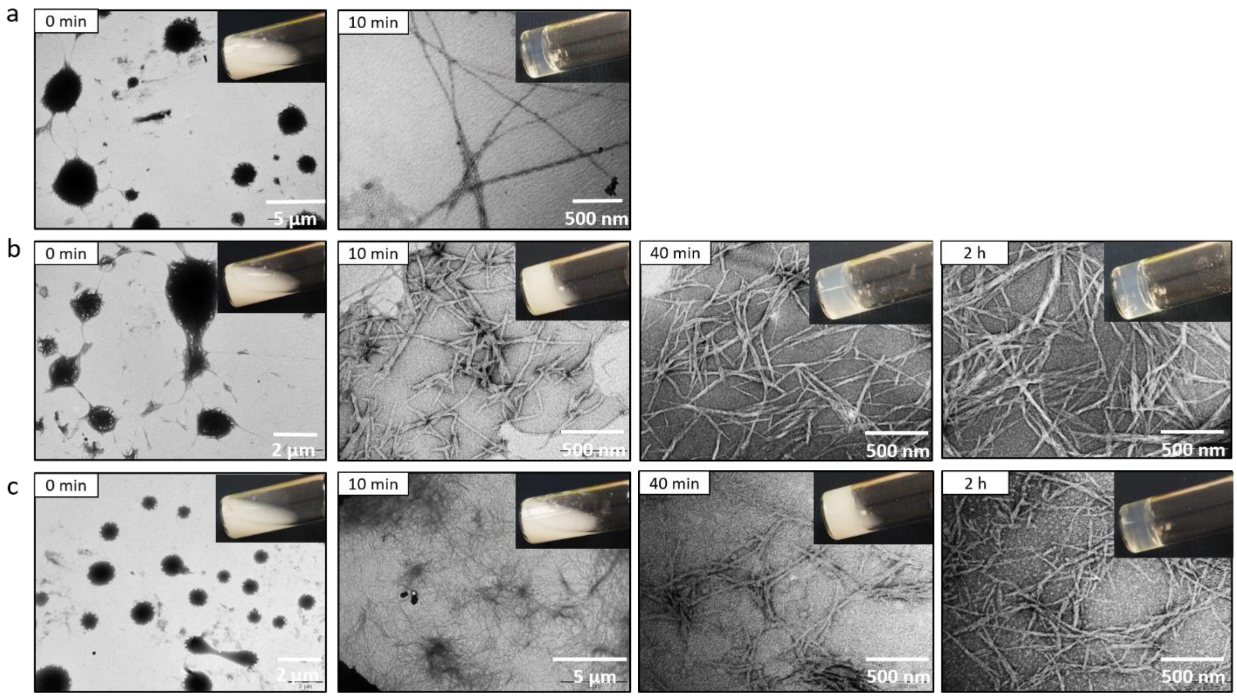

Figure S6 Gelation kinetics of Fmoc-Phe-Phe and the hybrid hydrogels. Time-lapse optical images and the corresponding TEM images of (a) Fmoc-Phe-Phe, (b) Fmoc-Phe-Phe/Fmoc-Gly-Pro-Hyp and (c) Fmoc-Phe-Phe/Fmoc-Gly-Pro-Pro for different time points.

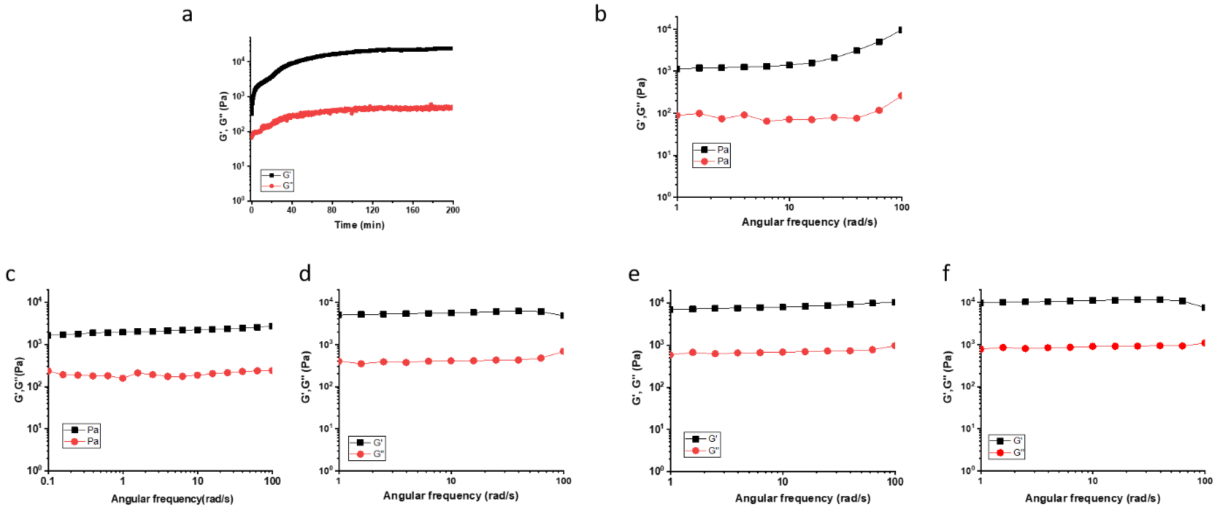

Figure S7 (a) Time sweep oscillation measurements of *in situ* hydrogel formation by Fmoc-Phe-Phe. (b-f) Dynamic frequency sweep experiments of (b) Fmoc-Phe-Phe, (c) Fmoc-Phe-Phe/Fmoc-Gly-Pro-Hyp, (d) Fmoc-Phe-Phe/Fmoc-Hyp-Pro-Gly, (e) Fmoc-Phe-Phe/Fmoc-Gly-Pro-Pro, and (f) Fmoc-Phe-Phe/Fmoc-Pro-Pro-Gly.

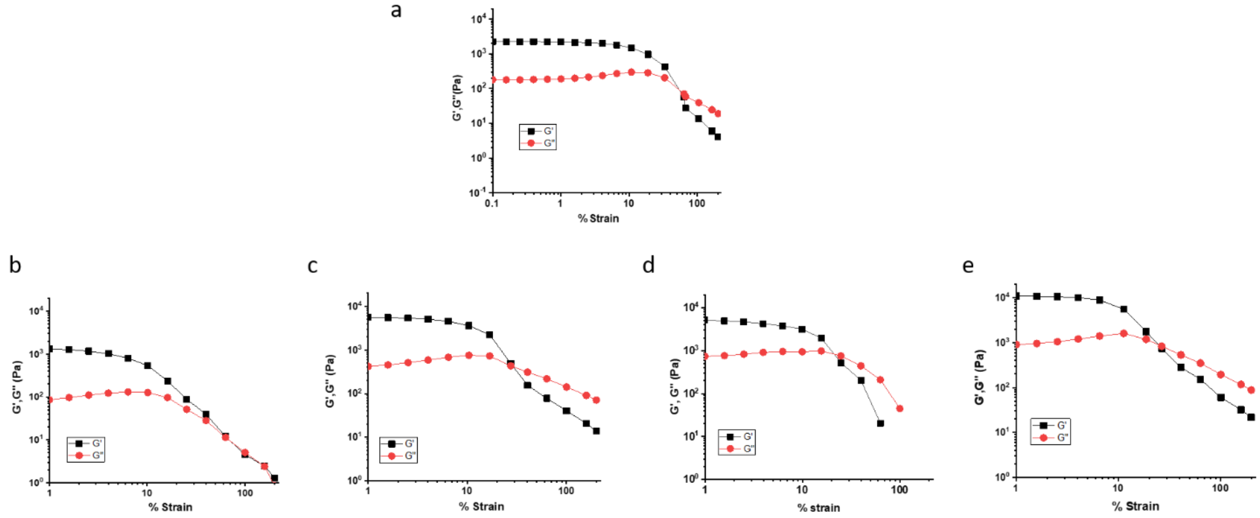

Figure S8 Dynamic strain sweep experiments (at 1 Hz frequency) of (a) Fmoc-Phe-Phe, (b) Fmoc-Phe-Phe/Fmoc-Gly-Pro-Hyp, (c) Fmoc-Phe-Phe/Fmoc-Hyp-Pro-Gly, (d) Fmoc-Phe-Phe/Fmoc-Gly-Pro-Pro, and (e) Fmoc-Phe-Phe/Fmoc-Pro-Pro-Gly.

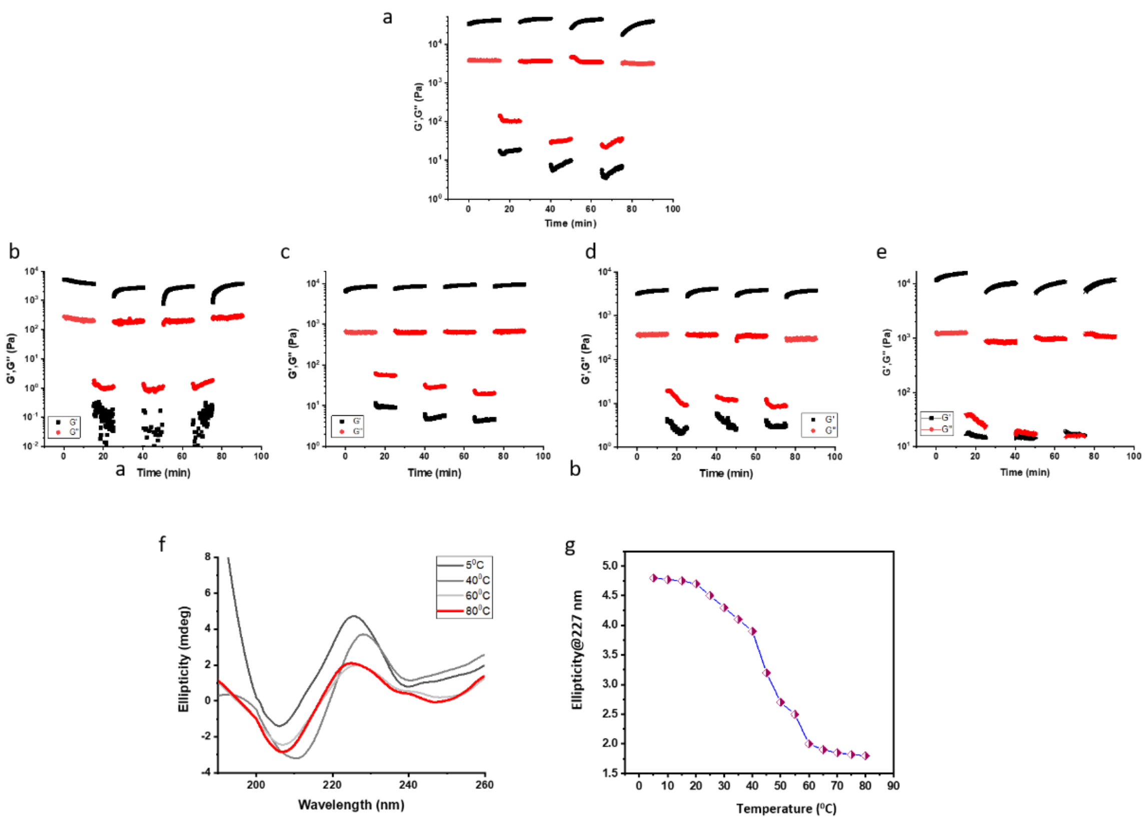

Figure S9 Recovery of the mechanical properties of the gels after shear deformation by step strain experiments of (a) Fmoc-Phe-Phe, (b) Fmoc-Phe-Phe/Fmoc-Gly-Pro-Hyp, (c) Fmoc-Phe-Phe/Fmoc-Hyp-Pro-Gly, (d) Fmoc-Phe-Phe/Fmoc-Gly-Pro-Pro, and (e) Fmoc-Phe-Phe/Fmoc-Pro-Pro-Gly. The hydrogels were subjected to a cycle oftime sweep experiments with periodic low (0.1%) and high (100%) strain values. (f,g) Temperature-dependent CD spectra of Fmoc-Phe-Phe/Fmoc-Pro-Pro-Gly. The ellipticity at 227 nm with respect to the temperature is demonstrated in (g).

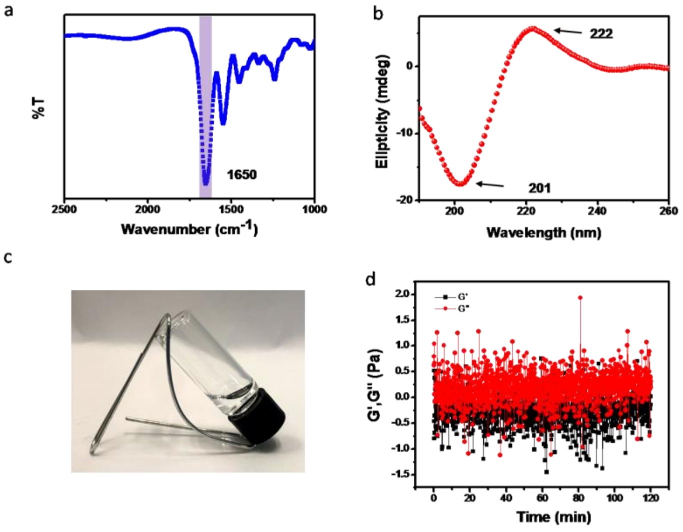

Figure S10 Characterization of native collagen. (a) FT-IR spectrum of collagen reveals distinct vibrational frequency at 1650 cm^{-1} in the amide I region, signature of polyproline II helical conformation, (b) CD spectrum of collagen showing a maximum at 222 nm and a minimum around 201 nm, the characteristics of a polyproline type II helix conformation. (c) Gelation demonstrated by tilted vial. (d) Time sweeps oscillation measurements.

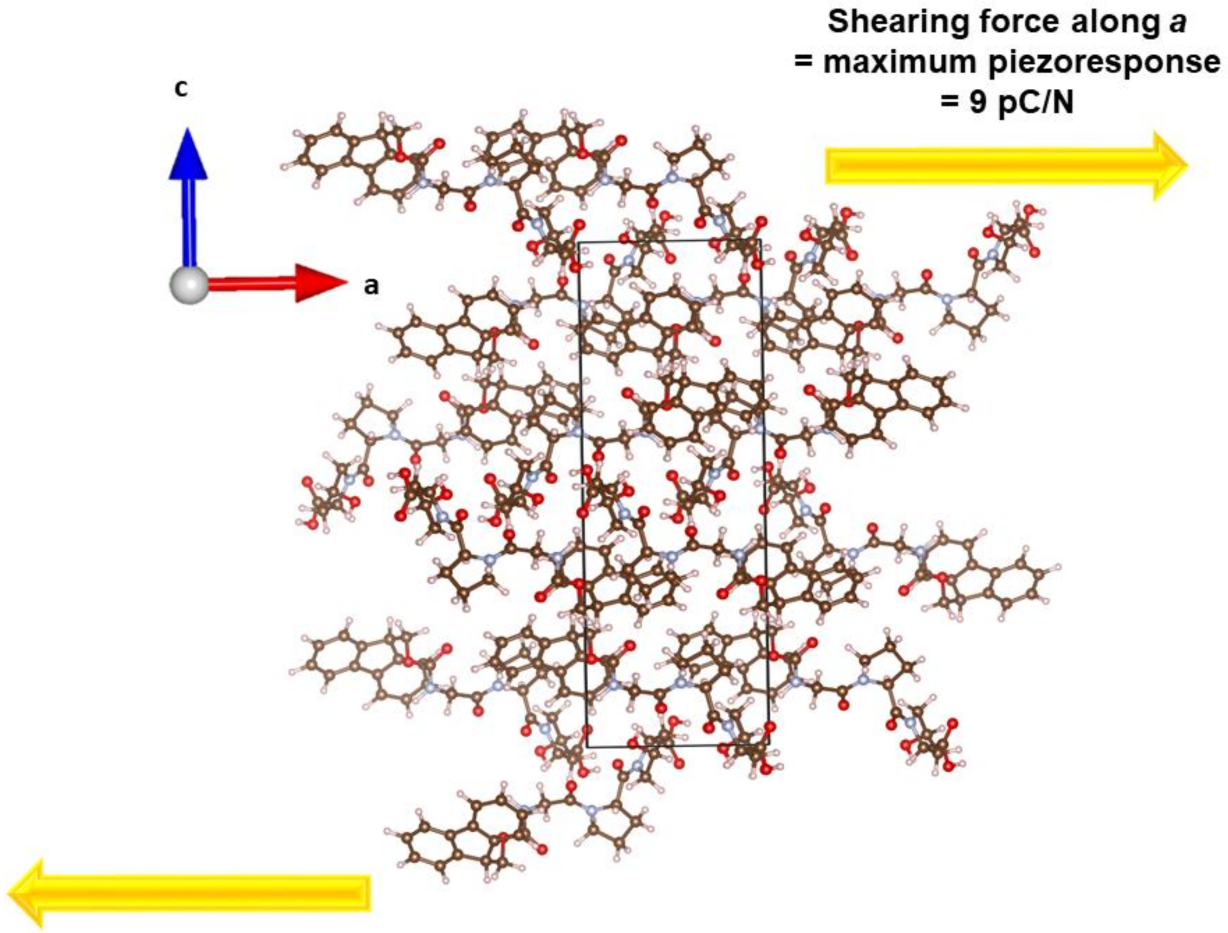

Figure S11 DFT calculations showing that shearing of the crystallographic *a* axis can induce a maximum piezoelectric response of 8.6 pC/N.

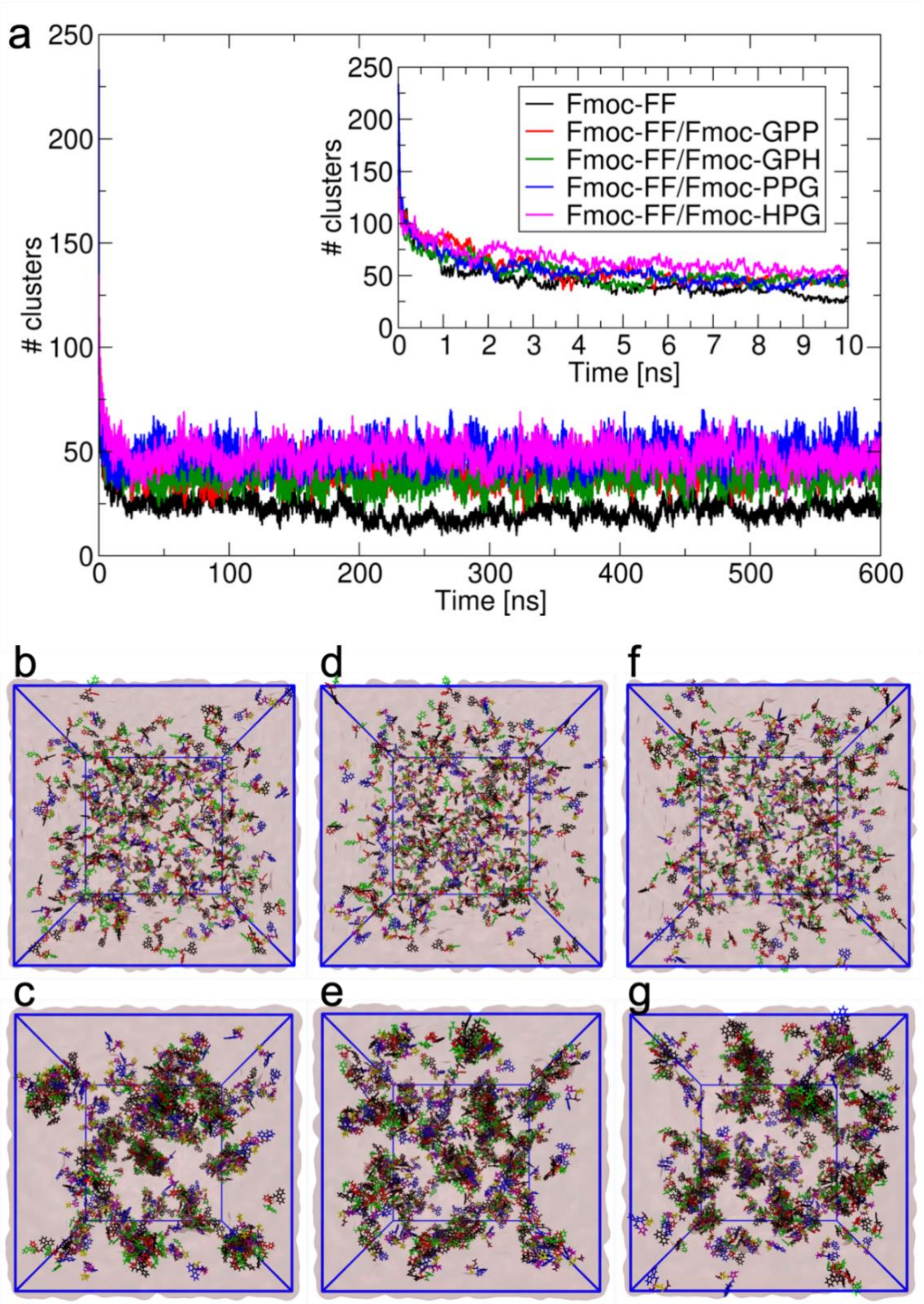

Figure S12 (a) The number of clusters formed as a function of time. The inset shows the first 10ns of the simulation. The raw data shown herein was used to construct the smoothed timelines in main text Figure 6. (b, d, f) The starting random and (c, e, g) final assembled structures of the (b, c) Fmoc-Phe-Phe/Fmoc-Gly-Pro-Pro co-assembly, (d, e) Fmoc-Phe-Phe/Fmoc-Pro-Pro-Gly co-assembly, and (f, g) Fmoc-Phe-Phe/Fmoc-Hyp-Pro-Gly co-assembly after 600 ns of equilibrated room temperature dynamics in water. Water is depicted by the transparent red/pink surface, peptides are depicted assticks. Fmoc of Fmoc-Phe-

Phe is colored in black with the first Phein red and the second Phe in green, the Fmoc of Fmoc-Gly-Pro-Hyp is colored in blue,Glyinbrown, Pro in magenta, and Hyp in yellow.

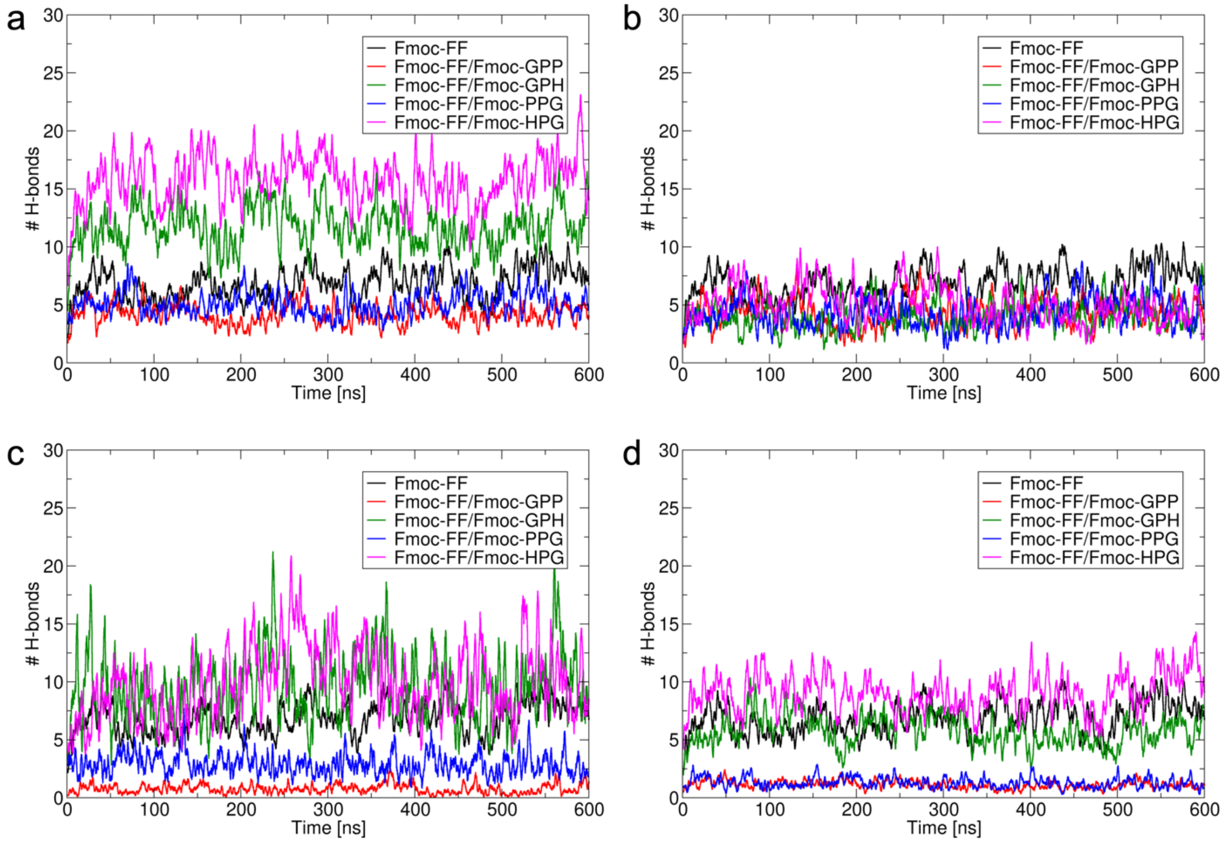

Figure S13 Running average (every 2 ns) of the number of hydrogen bonds (HBs) as a function of time. (a) Total number of HBs between all peptide molecule in the system. (b) Normalized number of HBs between Fmoc-Phe-Phe molecules. (c) Normalized number of HBs between co-peptide molecules. (d) Number of HBs between Fmoc-Phe-Phe and each of the co-peptides.

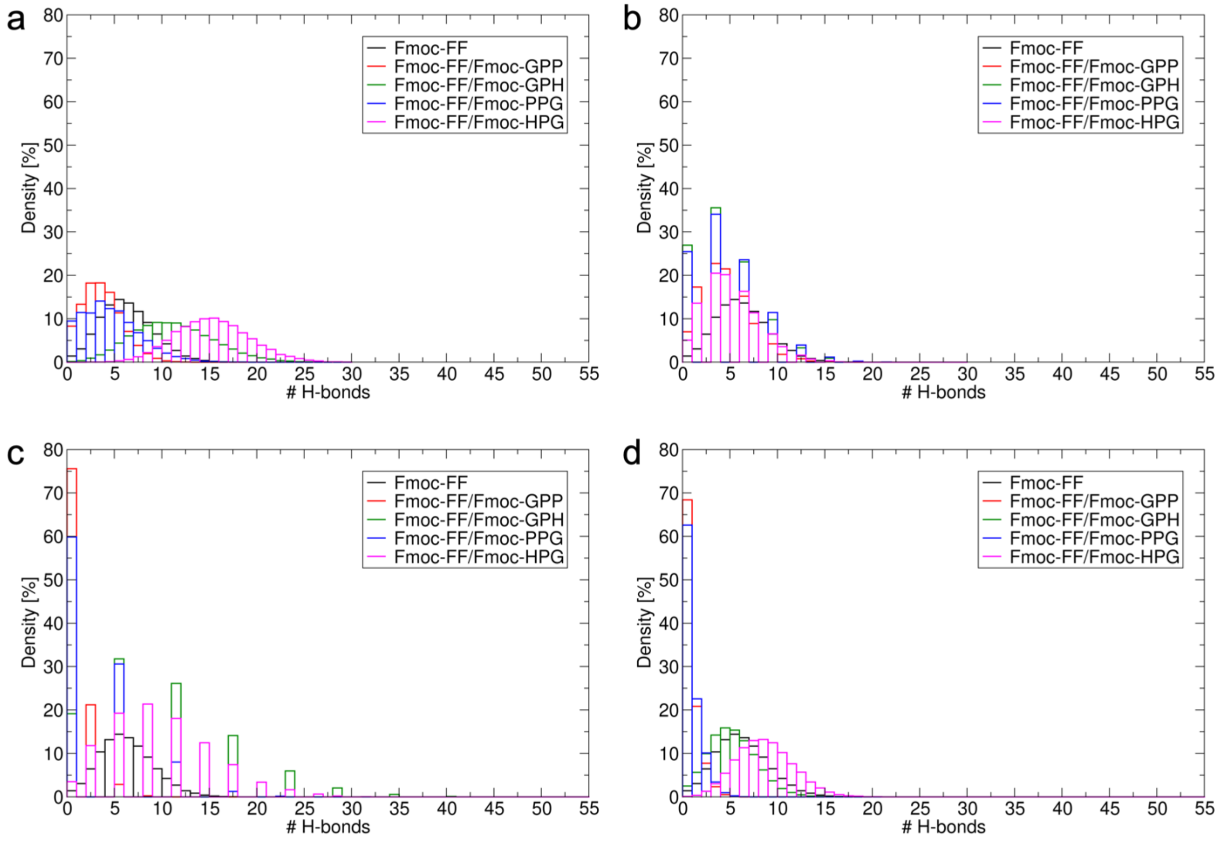

Figure S14 Distribution of the number of HBs. (a) HBs between all peptide molecule in the system. (b) HBs between Fmoc-Phe-Phe molecules. (c) HBs between co-peptide molecules. (d) HBs between Fmoc-Phe-Phe and each of the co-peptides.

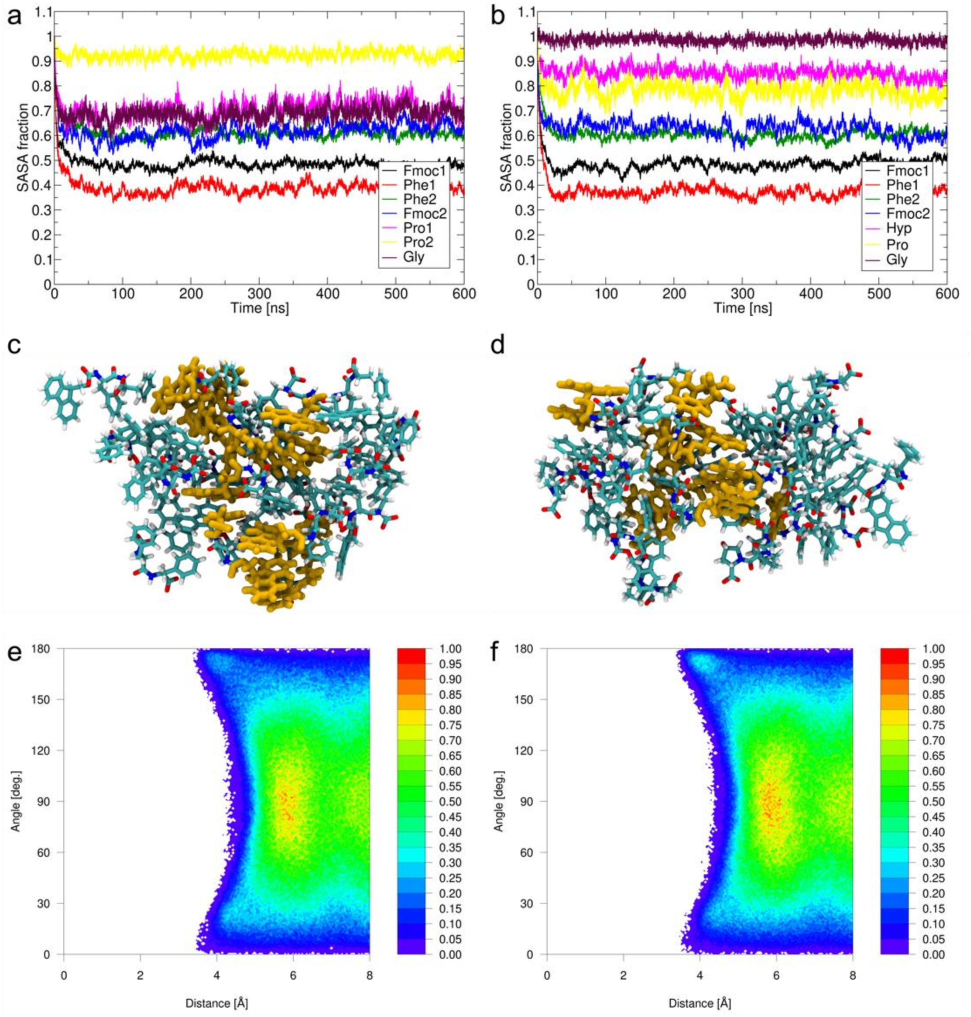

Figure S15 (a, b) Timeline of the SASA fraction of different groups for (a) Fmoc-Phe-Phe/Fmoc-Gly-Pro-Pro and (b) Fmoc-Phe-Phe/Fmoc-Hyp-Pro-Gly.(c, d) Representative supramolecular packing of (c) Fmoc-Phe-Phe self-assembly and (d) Fmoc-Phe-Phe/Fmoc-Gly-Pro-Hyp co-assembly. The π-π stacked sub-clusters are shown in yellow molecular representation. (e, f) Free energy landscape for each co-assembly mapped over the last 100 ns of the simulation. (e) Fmoc-Phe-Phe/Fmoc-Gly-Pro-Pro. (f) Fmoc-Phe-Phe/Fmoc-Hyp-Pro-Gly.

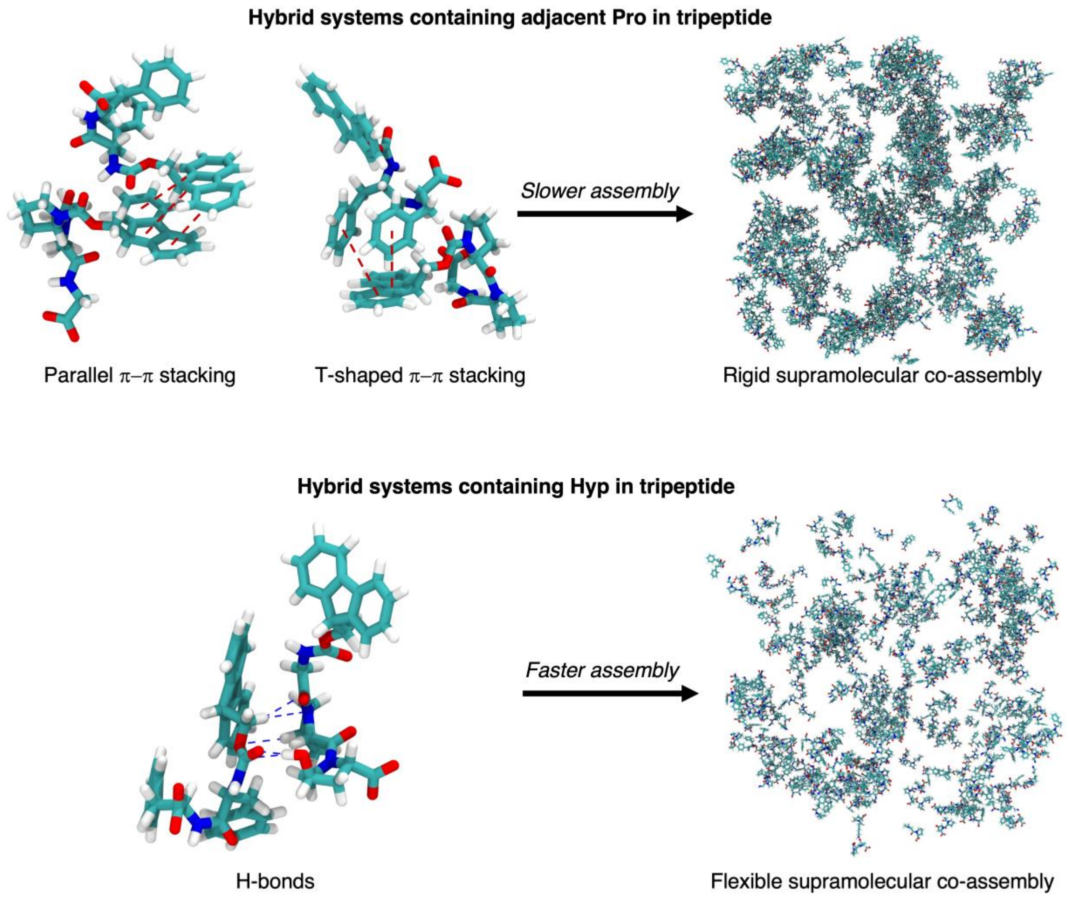

Figure S16 Schematic of self-assembly mechanism of hybrid systems with and without Hyp.

Supplementary references

(1) Fiolhais, C.;Nogueira,F.;Marques,M. A.A primer in density functional theory. Vol. 620 (Springer Science & Business Media, 2003).

(2) Hafner,J.Ab-Initio Simulations of Materials Using VASP: Density-Functional Theory and Beyond. *J.comput. chem.* **2008**,*29*, 2044-2078.

(3) Ernzerhof,M.;Scuseria,G. E.Assessment of the Perdew–Burke–Ernzerhof Exchange-Correlation Functional. *J. chem. phys.* **1999**,*110*, 5029.

(4) Grimme,S.;Antony,J.;Ehrlich,S.;Krieg,H.A Consistent and Accurate ab Initio Parametrization of Density Functional Dispersion Correction (DFT-D) for the 94 Elements H-Pu.*J. chem. phys.* **2010**,*132*, 154104.

(5) Kresse,G.;Joubert,D.From Ultrasoft Pseudopotentials to the Projector Augmented-Wave Method. *Phys. Rev. B***1999**,*59*, 1758-1775.

(6) Wu,X.;Vanderbilt,D.;Hamann,D.SystematicTreatment of Displacements, Strains, and Electric Fields in Density-Functional Perturbation Theory. *Phys. Rev. B***2005**,*72*, 035105.

(7) Chung,D.;Buessem,W. The Voigt-Reuss-Hill (VRH) Approximation and the Elastic Moduli of Polycrystalline ZnO, TiO2 (Rutile), and α-Al_2O_3. *J. Appl. Phys.***1968**,*39*, 2777.

(8) Hill,R.The Elastic Behaviour of a Crystalline Aggregate. *Proc. Phys. Soc. A***1952**,*65*, 349.

(9) Momma,K.;Izumi,F. VESTA 3 for Three-Dimensional Visualization of Crystal, Volumetric and Morphology Data. *J. Appl. Crystallogr*.**2011**,*44*, 1272-1276.

(10) Vanommeslaeghe,K.;MacKerell Jr.,A. D. Automation of the CHARMM General Force Field (CGenFF) I: Bond Perception and Atom Typing. *J. Chem. Inf. Model***2012**, *52*, 3144-3154.

(11) Vanommeslaeghe,K.;Raman, E. P.;MacKerell, Jr.,A. D.Automation of the CHARMM General Force Field (CGenFF) II: Assignment of Bonded Parameters and Partial Atomic Charges. *J. Chem. Inf. Model*2012, **52**, 3155-3168.

(12) Vanommeslaeghe, K.*et al.*,CHARMM General Force Field: A Force Field for Drug-Like Molecules Compatible with the CHARMM All-Atom Additive Biological Force Fields. *J. Comput. Chem.* **2010**,*31*, 671-690.

(13) Bayly, C. I.;Cieplak,P.;Cornell, W.;Kollman,P. A.A well-behaved electrostatic potential based method using charge restraints for deriving atomic charges: the RESP model.*J. Phys. Chem.* **1993**, *97*, 10269-10280.

(14) Gaussian 16 Rev. C.01 (Wallingford, CT, 2016).

(15) Wang,J.;Wang,W.;Kollman,P. A.;Case,D. A.Automatic Atom Type and Bond Type Perception in Molecular Mechanical Calculations. *J. Mol. Graph. Model***2006**, *25*, 247-260.

(16) Huang,J.*et al.*CHARMM36m: An Improved Force Field for Folded and Intrinsically Disordered Proteins. *Nat Methods***2017**, *14*, 71-73.

(17)Van Der Spoel, D.*et al.*GROMACS: Fast, Flexible, and Free. *J. Comput. Chem.* **2005**,*26*, 1701-1718.

(18) Hockney,R. W.Potential Calculation and Some Applications. *Methods Comput. Phys.***1970**,*9*, 136.

(19) Hess,B.;Bekker,H.;Berendsen,H. J.;Fraaije,J. G.LINCS: A Linear Constraint Solver for Molecular Simulations. *J. comput. chem.* **1997**,*18*, 1463-1472.

(20) Miyamoto,S.;Kollman, P. A.Settle: An Analytical Version of the SHAKE and RATTLE Algorithm for Rigid Water Models. *J. comput. chem.* **1992**,*13*, 952-962.

(21) Darden,T.;York,D.;Pedersen,L.Particle Mesh Ewald: An N·log(N) Method for Ewald Sums in Large Systems. *J. chem. phys.***1993**,*98*, 10089.

(22) Bussi,G.;Donadio,D.;Parrinello,M.Canonical Sampling Through Velocity Rescaling. *J. Chem. Phys.***2007**,*126*, 014101.

(23) Parrinello,M.;Rahman,A. Crystal Structure and Pair Potentials: A Molecular-Dynamics Study. *Phys. Rev. Lett.* **1980**, *45*, 1196.

(24) Humphrey, W.;Dalke,A.;Schulten,K.VMD: Visual Molecular Dynamics. *J. Mol. Graph.* **1996**,*14*, 33-38.
